# Genome-wide association study of seedling leaf rust resistance in European winter wheat cultivars

**DOI:** 10.1007/s13353-025-00976-2

**Published:** 2025-06-09

**Authors:** Paweł Cz. Czembor, Urszula Piechota, Jie Song, Dariusz Mańkowski, Magdalena Radecka-Janusik, Dominika Piaskowska, Piotr Słowacki, Andrzej Kilian

**Affiliations:** 1 Plant Breeding and Acclimatization Institute – National Research Institute, 05-870 Radzików, Poland; 2 Diversity Arrays Technology Pty Ltd, University of Canberra, Canberra, Australia

**Keywords:** Genome-wide association study, Leaf rust, Resistance, Wheat

## Abstract

Leaf rust, caused by *Puccinia triticina*, is a major wheat disease that impacts yield and quality. This study aimed to identify genetic loci associated with seedling resistance to leaf rust in European winter wheat cultivars. A genome-wide association study was conducted on a panel of 181 wheat genotypes, including 143 modern cultivars and 38 lines with known leaf rust resistance genes. Pathogen evaluation involved 18 *P. triticina* isolates, which revealed diverse virulence levels and allowed resistant cultivars to be identified. The study identified 88 marker–trait associations clustered into 23 quantitative trait loci (QTL) across 13 chromosomes. Three QTL—*QLr.ihar-1B.1*, *QLr.ihar-3D.1*, and *QLr.ihar-4 A.1*—correspond to the major resistance genes *Lr26*, *Lr24*, and *Lr28*. Several QTL appear novel, with six (*QLr.ihar-2B.2*, *QLr.ihar-3 A.1*, *QLr.ihar-3B.2*, *QLr.ihar-7 A.1*, *QLr.ihar-7D.1*, and *QLr.ihar-7D.2*) explaining over 20% of phenotypic variance that could be considered for breeding purposes. Among 113 resistant cultivars, only 23 QTL were present in 51 genotypes, suggesting that resistance in the remaining 62 cultivars is under control of unidentified loci. The findings highlight the complex and diverse resistance patterns in European wheat, offering significant insights for breeding programs targeting enhanced leaf rust resistance.

**Supplementary Information:**

The online version contains supplementary material available at 10.1007/s13353-025-00976-2.

## Introduction

Wheat leaf rust, caused by *Puccinia triticina*, is an important foliar disease of wheat (*Triticum aestivum* L.) worldwide, negatively affecting yield and grain quality (Bolton et al. 2008; Huerta-Espino et al. 2011). Breeding of resistant wheat cultivars is the most cost-effective and environmentally sound strategy to prevent these losses. In general, resistance has been classified into all-stage resistance (also known as seedling resistance) and adult plant resistance (APR) (Bolton et al. 2008). Seedling resistance is mostly conditioned by major genes conferring a high level of resistance and race specific. Conversely, APR is usually quantitatively inherited and the most effective at later stages of plant growth, reducing the disease infection progress but commonly race nonspecific (Bolton et al. 2008; Lagudah 2011). Continuous evolution of the *P. triticina* population, with new virulences, can in a relatively short time overcome resistance conditioned by major genes, but APR is considered to be more durable.

Over 80 leaf rust resistance genes (*Lr*) have been identified (McIntosh 2024). Most of these are major genes, which acquire significance in plant protection at the seedling stage, particularly in production environments conducive to early-season disease development (Huerta-Espino et al. 2011). Therefore, combining major resistance genes and APR within the same genotype may ensure greater and longer lasting disease resistance, and this strategy is considered one of the main components of informed deployment of resistance in cereals (Boyd et al. 2013; Burdon et al. 2014). Utilization of major resistance genes among well-adapted breeding lines/cultivars can take advantage of new sources of resistance originating from external gene pools (e.g., landraces and wild relatives). Especially in a context of breeding programs focusing on complex traits like yield, breeding for disease resistance can be hindered by linkage drag and competition drag (Summers and Brown 2013).

In order to efficiently apply major resistance genes in wheat breeding programs, information on major resistance genes carried by cultivars is required. Previously, identification of most resistance genes relied on bi-parental populations and linkage-mapping methods using low-resolution molecular marker systems. Advances in next-generation sequencing (NGS) technologies foster development of genotyping by sequencing-based (GBS) platforms, including the DArT-seq system (Diversity Arrays Technology, Canberra, Australia, https://www.diversityarrays.com/), which provides thousands of single nucleotide polymorphism (SNP) as well as SilicoDArT (presence/absence variations, PAV) markers. High-throughput genotyping technologies coupled with the improved statistical algorithms resulted in development of an alternative approach for mapping traits, known as genome-wide association studies (GWAS) or association mapping (AM) (Gupta et al. 2005). GWAS use linkage disequilibrium (LD) between alleles within a set of diverse genotypes to identify markers associated with a phenotypic trait. This mapping approach takes advantage of historical recombination events that have accumulated over a number of generations in populations. Recombination leads to break-up of the LD blocks within the genome and causes faster decay of the LD in the AM panels compared to bi-parental populations with a limited number of recombination events and small allelic diversity represented only by the parents. This fundamental difference allows GWAS to identify associated loci with the phenotypic trait at a much higher resolution (Flint-Garcia et al. 2003; Gupta et al. 2005; Mackay and Powell 2007). However, population structure and genetic relatedness among individuals can lead to spurious associations between markers and traits. To address the challenges in genetic analysis, several methodologies have been developed, among which the linear mixed model (LMM) is one of the most widely employed. The LMM effectively accounts for population structure (Q) and kinship (K), allowing for a more accurate assessment of genetic variation and relatedness among individuals (Myles et al. 2009). Notable advancements to LMMs include optimized variance component estimation, which enhances the reliability of the inferences drawn from the model (Runcie & Crawford 2019), as well as improvements in genetic prediction accuracy (Maier et al. 2018; Lloyd-Jones et al. 2019). Furthermore, these models have been refined to better capture the intricate interrelationships of complex traits (Ge et al. 2022). Despite these improvements, the LMM-Q + K framework remains a widely accepted and effective approach for analyzing datasets with relatively straightforward structures, such as those utilized in our study.

Association mapping has been conducted for leaf rust resistance on seedlings and/or at the adult plant stage in common wheat (*T. aestivum*) using landraces or old cultivars (Kertho et al. 2015; Kankwatsa et al. 2017; Pasam et al. 2017), synthetic wheat (Jighly et al. 2016), breeding lines (Gao et al. 2016; Juliana et al. 2018), or using mixed panels (Turner et al. 2017; Gerard et al. 2018; Sapkota et al. 2019; Iqbal et al. 2022; Delfan et al. 2023; Kaur et al. 2023). In most cases, studies examined a geographically diverse panel of wheat that also included cultivars and breeding lines from Europe (Turner et al. 2017; Gerard et al. 2018; Kaur et al. 2023) or investigated a relatively small set of European genotypes representing a limited genetic pool (Tyrka et al. 2017). However, there is still limited information on genetic architecture underlying leaf rust resistance specifically in currently grown European winter wheat cultivars. This study aims to fill this knowledge gap by identifying leaf rust resistance genes present in commercial European winter wheat cultivars using GWAS. The results of this research provide breeders with valuable genetic insights to optimize resistance gene deployment in European wheat breeding programs. Such precise knowledge of resistance loci distribution and their frequency in modern cultivars, coupled with information on the current virulence structure of pathogen populations, is essential for the strategic breeding of cultivars with effective and durable resistance.

## Materials and methods

### Plant materials

A set of 143 European winter wheat cultivars (Table S1) and differential set comprising 38 lines with known leaf rust resistance genes (Table S2) were used to establish a leaf rust association mapping (AM) panel (181 genotypes in total). The main criterion for choosing wheat cultivars was usually medium to high expression of resistance to leaf rust noticed in varieties registered in national official trials in Poland (COBORU 2013), Germany (Bundessortenamt 2013), France (GEVES 2013), Austria (AGES 2014), or other sources for the UK (Bayles 2013). A set of differential lines with known *Lr* genes assisted in gene postulations.

### Pathogen and disease evaluation

From the pathogen collection held at PBAI-NRI (Radzików, Poland), 18 *P. triticina* isolates (Pt1–Pt18) were selected (Table 1). These isolates represented diverse arrays of virulence and avirulence to seedling resistance genes.

**Table 1:** Virulence-avirulence spectra of *Puccinia triticina* isolates used in association mapping for leaf rust resistance genes

Isolate	Name	Virulence combination (ineffective *Lr* genes)	Avirulent on *Lr* genes	Race^1^	Country^2^	Host	Year of collection	Location
Pt1	Pt 2902	1, 3, 3bg, 3ka, 10, 11, 14a, 14b, 15, 16, 17, 18, 20, 23, 26, 30, 32, 33, 36, 44, 27 + 31, LrB(Carina), LrB(PI268316)	2a, 2b, 2c, 9, 19, 21, 24, 25, 28, 29, 38, 52, 63, 64	MHTT	PL	Wheat	Unknown	Unknown
Pt2	NIAB 06–23	1, 3, 3bg, 3ka, 10, 11, 14a, 14b, 15, 16, 17, 18, 21, 23, 26, 27 + 31, 30, 33, 36, 44, 64	2a, 2b, 2c, 9, 19, 20, 24, 25, 28, 29, 32, 38, 52, 63, LrB(Carina), LrB(PI268316)	MHTK	UK	Wheat	Unknown	Unknown
Pt3	Pt 1602	2c, 3, 3bg, 3ka, 10, 11, 14a, 14b, 15, 16, 17, 18, 20, 21, 23, 26, 27 + 31, 30, 32, 33, 36, 38, 44, 63, LrB(Carina), LrB(PI268316)	1, 2a, 2b, 9, 19, 24, 25, 28, 29, 52, 64	FHTT	PL	Wheat	Unknown	Unknown
Pt4	NIAB 06–94	2a, 2b, 2c, 10, 11, 14a, 14b, 16, 18, 20, 21, 23, 27 + 31, 28, 32, 33, 36, 38, 44, LrB(Carina), LrB(PI268316)	1, 3, 3bg, 3ka, 9, 15, 17, 19, 24, 25, 26, 29, 30, 52, 63, 64	JGGT	UK	Wheat	Unknown	Unknown
Pt5	Pt 1002	1, 3, 3bg, 3ka, 10, 11, 14a, 14b, 15, 17, 18, 20, 23, 26, 27 + 31, 30, 33, 36, 44, LrB(Carina), LrB(PI268316)	2a, 2b, 2c, 9, 16, 19, 21, 24, 25, 28, 29, 32, 38, 52, 63, 64	MCTT	PL	Wheat	Unknown	Unknown
Pt6	NIAB 06–98	2a, 2b, 2c, 3, 3bg, 3ka, 10, 11, 14a, 14b, 16, 17, 18, 20, 21, 25, 26, 27 + 31, 28, 29, 30, 32, 33, 36, 44, 63, 64, LrB(Carina), LrB(PI268316)	1, 9, 15, 19, 23, 24, 38, 52	KHTT	UK	Wheat	Unknown	Unknown
Pt7	Tr11_A1/12	2c, 3ka, 10, 11, 14a, 14b, 16, 17, 18, 21, 23, 24, 27 + 31, 30, 33, 44, 64, LrB(Carina), LrB(PI268316)	1, 2a, 2b, 3, 3bg, 9, 15, 19, 20, 25, 26, 28, 29, 32, 36, 38, 52, 63	DJTT	PL	Triticale	June 2012	Małyszyn
Pt8	Tr09_3/13	2c, 3, 3bg, 3ka, 10, 11, 14a, 14b, 15, 16, 17, 18, 21, 23, 24, 26, 27 + 31, 30, 32, 33, 36, 38, 44, 52, 63, 64, LrB(Carina), LrB(PI268316)	1, 2a, 2b, 9, 19, 20, 25, 28, 29	FKTT	PL	Triticale	June 2013	Kraków
Pt9	Tr09_8/13	2a, 2b, 2c, 3, 3ka, 10, 11, 14a, 14b, 15, 16, 17, 18, 21, 23, 24, 27 + 31, 30, 33, 44, 52, 64, LrB(Carina)	1, 3bg, 9, 19, 20, 25, 26, 28, 29, 32, 36, 38, 63, LrB(PI268316)	KJTT	PL	Triticale	June 2013	Kraków
Pt10	Tr11_2/13	2a, 2b, 2c, 3, 10, 11, 14a, 14b, 18, 21, 23, 24, 27 + 31, 29, 30, 32, 33, 38, 44, 52, 64, LrB(Carina), LrB(PI268316)	1, 3bg, 3ka, 9, 15, 16, 17, 19, 20, 25, 26, 28, 36, 63	KDHT	PL	Triticale	June 2013	Małyszyn
Pt11	Tr11_4/13	3, 2b, 2c, 10, 11, 14a, 14b, 15, 16, 17, 18, 23, 24, 27 + 31, 29, 30, 32, 33, 36, 44, 52, 64, LrB(Carina)	1, 2a, 3bg, 3ka, 9, 19, 20, 21, 25, 26, 28, 38, 63, LrB(PI268316)	FJKT	PL	Triticale	June 2013	Małyszyn
Pt12	Tr10_2/12	2b, 2c, 3, 3ka, 10, 11, 14a, 14b, 15, 16, 17, 18, 21, 23, 24, 27 + 31, 29, 30, 32, 33, 38, 44, 63, 64, LrB(Carina), LrB(PI268316)	1, 2a, 3bg, 9, 19, 20, 25, 26, 28, 36, 52	FJTT	PL	Triticale	June 2012	Krzeczowice
Pt13	11_16/15	2b, 2c, 3ka, 10, 11, 14a, 14b, 18, 25, 30, 32, 33, 38, 44, 64, LrB(Carina)	1, 2a, 3, 3bg, 9, 15, 16, 17, 19, 20, 21, 23, 24, 26, 27 + 31, 28, 29, 36, 52, 63, LrB(PI268316)	DBRT	PL	Triticale	June 2015	Małyszyn
Pt14	10_14/15	1, 3bg, 3ka, 10, 11, 14a, 14b, 15, 16, 17, 18, 20, 27 + 31, 30, 33, 36, 38, 44, LrB(Carina), LrB(PI268316)	2a, 2b, 2c, 3, 9, 19, 21, 23, 24, 25, 26, 28, 29, 32, 52, 63, 64	LGTT	PL	Triticale	June 2015	Krzeczowice
Pt15	10_2/15	1, 2c, 3bg, 3ka, 3, 10, 11, 14a, 14b, 16, 18, 21, 23, 26, 30, 33, 38, 44, 63, LrB(Carina)	2a, 2b, 9, 15, 17, 19, 20, 24, 25, 27 + 31, 28, 29, 32, 36, 52, 64, LrB(PI268316)	PHRT	PL	Triticale	June 2015	Krzeczowice
Pt16	9_4/15	1, 3, 3bg, 3ka, 10, 11, 14a, 14b, 15, 16, 17, 18, 21, 23, 26, 27 + 31, 30, 32, 33, 36, 38, 44, 63, LrB(Carina), LrB(PI268316)	2a, 2b, 2c, 9, 19, 20, 24, 25, 28, 29, 52, 64	MHTT	PL	Triticale	June 2015	Kraków
Pt17	P14-88–2	2c, 3, 3bg, 3ka, 10, 11, 14a, 14b, 16, 17, 18, 20, 21, 27 + 31, 30, 33, 38, LrB(Carina), LrB(PI268316)	1, 2a, 2b, 9, 15, 19, 23, 24, 25, 26, 28, 29, 32, 36, 44, 52, 63, 64	FGTT	PL	Wheat	June 2014	Krzeczowice
Pt18	Pt13/10–78-2	1, 3ka, 10, 14a, 14b, 15, 16, 17, 18, 21, 25, 27 + 31, 30, 32, 33, 36, 38, 44, 64, LrB(Carina), LrB(PI268316)	2a, 2b, 2c, 3, 3bg, 9, 11, 19, 20, 23, 24, 26, 28, 29, 52, 63	LGTT	PL	Triticale	June 2013	Krzeczowice

^1^The North American system of race nomenclature (Long and Kolmer 1989)

^2^Country designation: PL—Poland; UK—United Kingdom

Each isolate was tested on the AM panel at the seedling stage under the controlled environment of a plant growth chamber. For each genotype, 3–5 seeds were sown in a horticulture tray and grown under 19 °C/15 °C (16-h day/8-h night) conditions. Isolate inoculations were done when the first leaf was fully developed (BBCH phase 11) using freshly multiplied urediniospores suspended in a carrier solvent, methoxy-nonafluorobutane (3 M Novec 7100 Engineered Fluid; 3 M Electronics Materials Solutions Division, St. Paul, USA) at 100 mg of spores per 100 ml of carrier Novec 7100 per two horticulture trays (each 60 × 30 × 4 cm deep with 104 cells). Inoculated seedlings were incubated overnight at 19 °C and 100% relative humidity provided by ultrasonic humidifiers. Thereafter, plant growth chamber conditions were changed to a 23 °C/19 °C (16-h day/8-h night) regime for further plant growth and disease development.

Leaf rust infection types (ITs) were assessed at 10–12 days post-inoculation using a 0–4 scale (Long and Kolmer 1989; McIntosh et al. 1995) where IT 0 = no visible symptoms, 0; = hypersensitive flecks, 1 = small uredinia with necrosis, 2 = small- to medium-size uredinia surrounded by chlorosis, 3 = medium-size uredinia with no chlorosis or necrosis, and 4 = large uredinia with no necrosis or chlorosis. Heterogeneous infection type evenly distributed over the leaf surface was designated as X (mesothetic reaction). Accessions with ITs of 0 to 2 and X were considered resistant, while scores of 3 and 4 were considered susceptible (McIntosh et al. 1995; Long and Kolmer 1989). Race designation was assigned according to the North American system of nomenclature (Long and Kolmer 1989).

### Genotyping, population structure, and linkage disequilibrium analysis

Genomic DNA was extracted from fresh seedling leaves collected from each genotype (100 mg) using a DNeasy Plant Mini Kit (Qiagen GmbH, Hilden, Germany). DNA samples were sent to Diversity Arrays Technology (DArT) Pty Ltd, Australia (http://www.diversityarrays.com) as a commercial service provider for whole-genome scan using the wheat DArTseq (1.0) platform. The full description of the DArTseq procedure was previously given by Sehgal et al. (2015). The physical positions of the markers were provided by DArT based on the Wheat_ChineseSpring04 reference genome.

Population structure (Q) was analyzed using a model-based clustering method using STRUCTURE v2.3.4 program (Pritchard et al. 2000) and principal coordinates analysis (PCoA) using Genstat v19.1.0.21390 (VSN International 2017). Only SNP markers (without those containing double null/null allele homozygote) were used to population structure analysis, and 25 independent runs were performed for a specified number of groups, *k*, from 1 to 10, with 20,000 burn-in length and 40,000 Markov chain Monte Carlo (MCMC) iterations. Number of groups (*k*) was determined based on the Δ*k* method (Evanno et al. 2005) deployed in the web-based program CLUMPAK (Kopelman et al. 2015). PCoA was conducted by applying a genotype kinship matrix calculated using nonredundant SNP markers and filtering out those with minor allele frequency less than 5 and 20% or more missing data (Genstat v19.1.0.21390). The PCoA plot was color coded according to groups revealed by STRUCTURE.

Linkage disequilibrium (LD) was estimated as the correlation coefficient (*r*^2^) between markers across the chromosomes and was plotted against the physical distance in base pairs (R package converted to KDCompute plugin developed by DArT Pty Ltd).

#### Genome-wide association study

Genome-wide association mapping applied the mixed linear model (MLM) (Yu et al. 2006) accounting for population structure using the R package algorithm rrBLUP implemented through the KDCompute plugin (DArT Pty Ltd.). The model is expressed as *y* = *Xβ* + *Zg* + *Sτ* + *ε*, where *X* is the design matrix relating to fixed effects of the entry (g), *Z* is the incidence matrix relating to genetic background, *S* is the incidence matrix relating to additive SNP and fixed effects, *y* is the vector of phenotypes, and *β* is a vector of fixed effects that can model both environmental factors and population structure. The variable *g* models the genetic background of each line as a random effect with *Var* [*g*] = *Kσ*^2^. The variable *τ* models the additive SNP effect as a fixed effect. The residual variance is *Var* [*ε*] = *Iσ*_*e*_^2^. For QTL designation, a *p*-value adjusted by the *q*-value for the false discovery rate (FDR) with a cut-off of 0.01 (–log(*q*-value) = 2.0) was necessary (Storey et al. 2020).

## QTL region detection and correspondence to known *Lr* genes

For a single marker associated with reaction to a certain *Pt* isolate, a –log(*q*-value) ≥ 2.0 for markers was required. The value of *r*^2^ ≥ 0.2 from LD analysis and physical distances were applied for assignment of cosegregating or adjacent markers into a single LD block representing a unique interval for trait-associated regions (MTA). The intervals whose physical positions overlapped were collapsed into single QTL. The QTL could contain one or several MTA with overlapping positions, but the most significant marker (–log(*q*-value)) with the largest determination coefficient value (*R*^2^) (Konigorski et al. 2020) was selected to represent the QTL.

Gene postulation was based on correspondence of both phenotype and genotype for *Lr* genes harbored by the differential set and the cultivar panel. A major resistance gene was identified if a significant marker was detected in the genome of only one line carrying a known seedling resistance *Lr* gene from among lines comprising the differential set and the same marker was found in the other cultivars. The marker location in the genome of the differential line was checked (absence/presence) against the known chromosomal location of the carried *Lr* gene (McIntosh 2024). Furthermore, both the differential line and cultivar had to be resistant to the *Pt* isolate used for the declared MTA. Additionally, potential candidate genes were inferred based on their closest proximity to the most significant marker, using the dataset deposited in BioMart (Ensembl Genomes release 60—October 2024).

## Results

### Pathogenicity of *P. triticina* isolates and seedling disease resistance

**Fig. 1:**
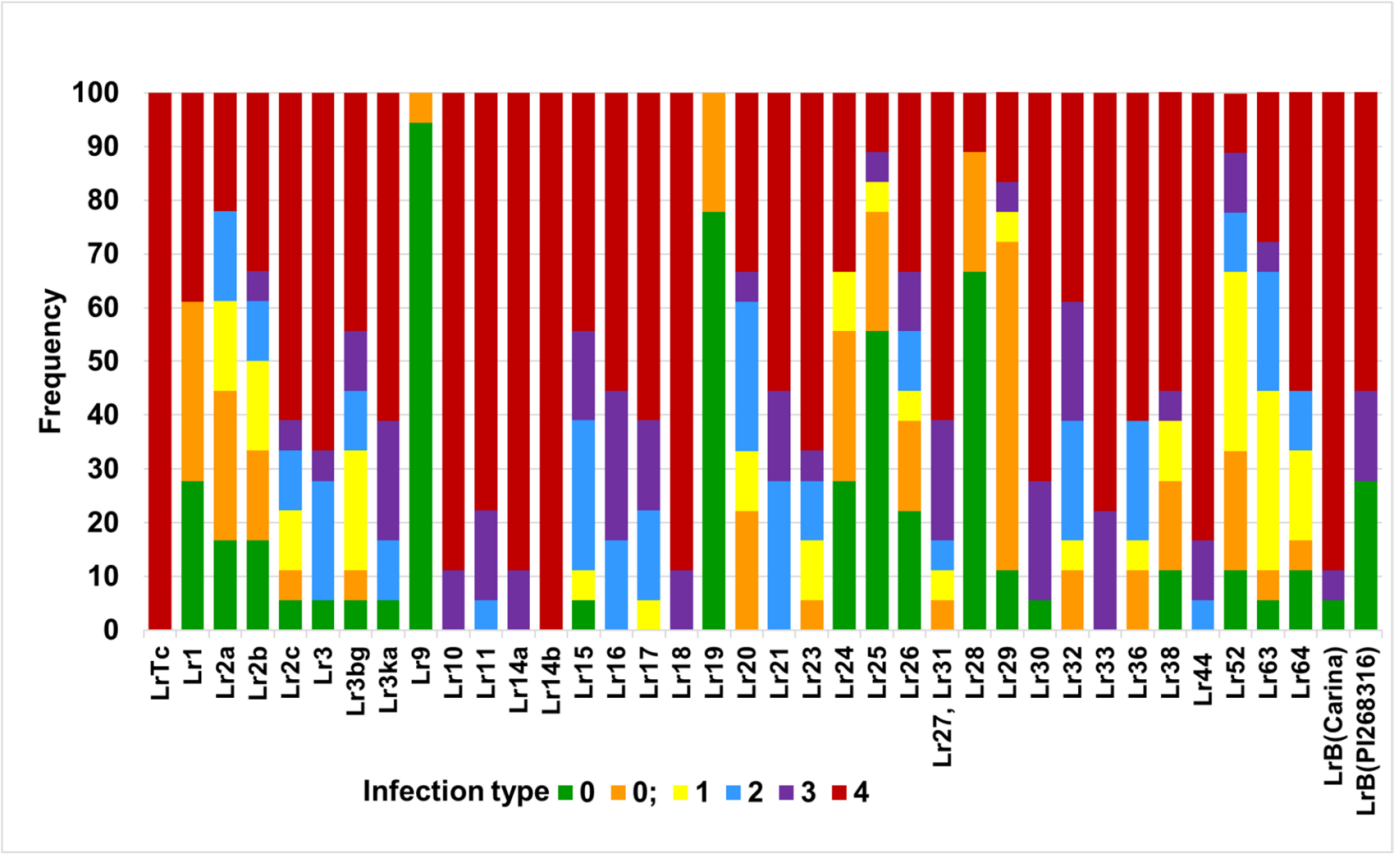
Profiles of infection types according to 0–4 scale (Long and Kolmer 1989; McIntosh et al. 1995) of wheat seedlings possessing known *Lr* genes against set of 18 *P. triticina* isolates

The set of 18 isolates of *P. triticina* represented diverse pathogenicity and lines with known *Lr* resistance genes had a different IT profile for all isolates (Fig. 1). However, the line with the resistance gene *Lr14b* was completely susceptible to all tested *Pt* isolates (IT = 4), similarly to that observed for the reference susceptible cultivar Thatcher. Also, for lines with *Lr10*, *Lr14a*, and *Lr18*, a compatible interaction was observed for all *Pt* isolates with IT scores of 3 and 4. None of the isolates was avirulent to *Lr33*, and IT for four isolates was 3 while for the others IT = 4 (Fig. 1). Avirulence (IT scores 0–2) for only one *Pt* isolate was observed for lines with *Lr11*, *Lr30*, *Lr44*, and *LrB*(Carina). Lines with genes *Lr9* and *Lr19* were resistant to all *Pt* isolates (IT scores 0 and 0;). All the other lines from the differential set expressed a wide range of IT scores for different numbers of isolates (Fig. 1).

## Resistance of wheat panel to *P. triticina* isolates

Out of 143 cultivars, 30 were found to be susceptible to all 18 isolates (Table S3). In contrast, nine cultivars demonstrated broad-spectrum resistance, exhibiting resistance to all 18 isolates: Capone, Caroll, Desamo, Lear, Lithium, Memory, Tentation, Waxy, and Xantippe. These cultivars represent 11 different resistance loci, with each cultivar possessing between 1 and 6 resistance genes, and no common pattern of resistance loci was observed. The study identified 113 cultivars that were resistant to at least one isolate. Furthermore, 29 cultivars showed resistance to at least half (nine or more) of the tested isolates (Table S3).

The most virulent isolate Pt4 was able to infect 122 out of 143 cultivars (85.3%). Conversely, the least virulent isolate, Pt7, could only infect 62 cultivars (43.4%). Other isolates displayed varying degrees of virulence: Pt1, Pt2, Pt3, Pt5, and Pt6 showed high virulence, infecting 70–80% of the cultivars; Pt8, Pt14, Pt16, and Pt18 exhibited moderate virulence, affecting 60–70% of the wheat lines; Pt9, Pt10, Pt11, Pt12, Pt13, Pt15, and Pt17 demonstrated lower virulence, infecting 50–60% of the cultivars (Table S3).

## Population structure analysis

**Fig. 2:**
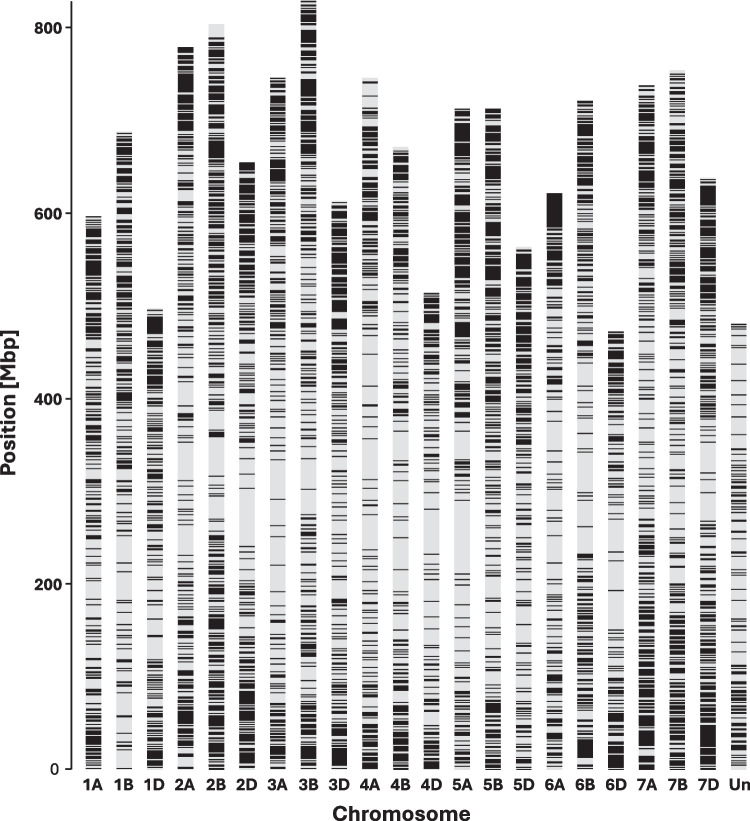
Physical mapping of 10,222 DArTSNP markers used for population structure analysis

**Fig. 3:**
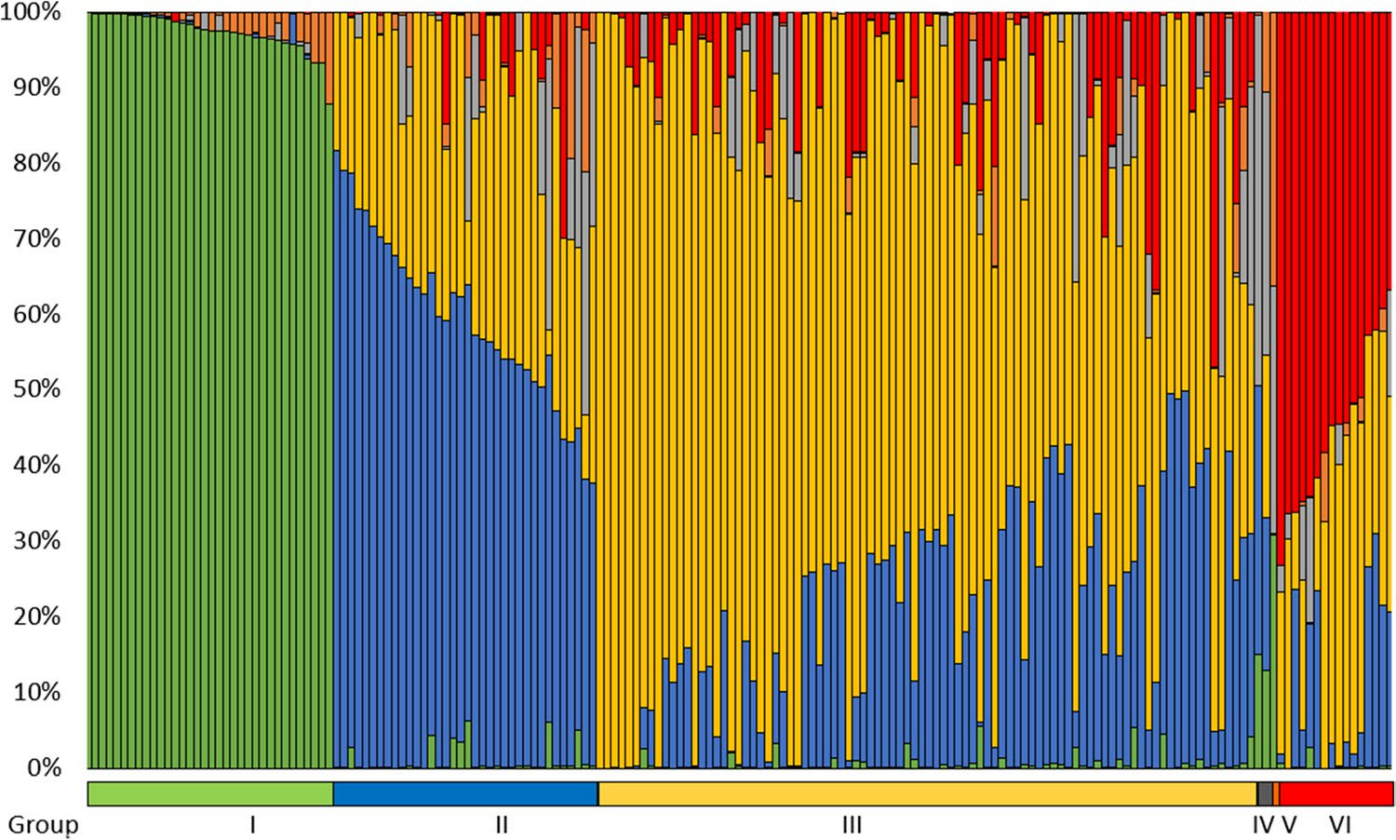
Population structure bar plot of ancestry proportion of the panel of 143 wheat cultivars identified through the analysis via the STRUCTURE program. Each genotype is represented by a vertical line divided into colored segments; the lengths and color of each individual indicate the proportions of the genome attributed to each subpopulation

**Fig. 4:**
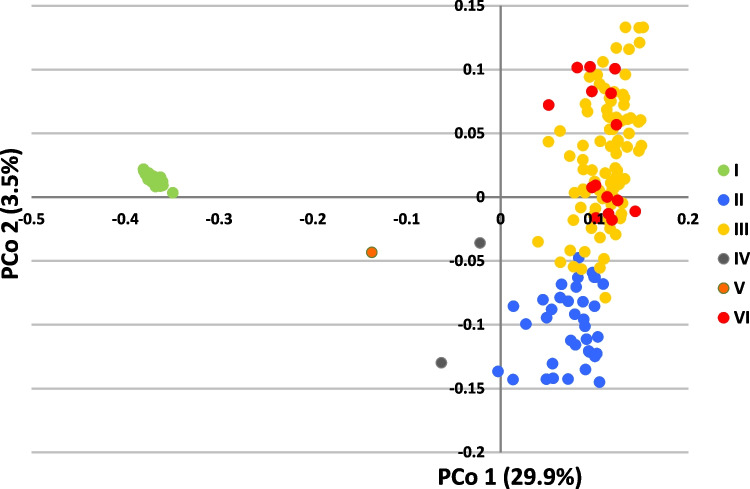
Scatterplot of the first two principal components (PCo1 and PCo2) of the panel of 143 wheat cultivars. Colors correspond to six groups determined in the population structure analysis via STRUCURE software (Fig. 3)

A total of 40,035 DArTSNP and 37,691 SilicoDArT markers were obtained from the DArTseq analysis. The marker name is composed of the core ID of the DArTseq clone and supplemented after the underscore symbol with the SNP position in the clone sequence. Due to low quality of DArTseq data, two differential lines with *Lr11* and *Lr44* genes were removed from further analyses. Physical location was assigned to 32,770 and 25,148 for DArTSNP and SilicoDArT markers, respectively. DArTSNP markers were represented in A, B, and D genomes by 32.6, 38.4, and 29.0%, respectively. SilicoDArT markers were distributed among A, B, and D genomes with the percentages 33.0, 43.6, and 23.4%, respectively. The physical positions of the selected 10,222 DArTSNP markers (only polymorphic and those with 100% complete data) on wheat chromosomes (Fig. 2) demonstrate a non-uniform distribution, showing greater marker density on distal chromosome regions compared to internal, pericentromeric areas. These markers were used in population structure analysis. The optimal number of *k* groups was determined to be 6, with Δ*k* = 42.7, and the other tested *k* values did not exceed 3.3. The six groups determined in the clustering analysis using the STRUCTURE (Table S4, Fig. 3) comprised group I (34 lines) consisting entirely of Thatcher NILs with known *Lr* resistance genes; groups II (36 cultivars), III (90 cultivars), and VI (16 cultivars), ranging over all cultivars but one, MV Lucilla, which together with line LrB(PI268316) formed IV group. The single genotype (*Lr* differential line) Gatcher emerged as a separate group, V. The groups comprising cultivars II, III, and VI did not cluster based on origin from a specific country or breeding company. In the PCoA, the first two principal coordinates explained 29.9 and 3.5% of variance, respectively (Fig. 4). The variation captured revealed distinct groups of genotypes very similar to that determined by the model-based clustering method (Fig. 3). However, groups III and VI overlapped on the graph of PCoA (Fig. 4).

## Marker–trait associations and QTL detection

**Fig. 5:**
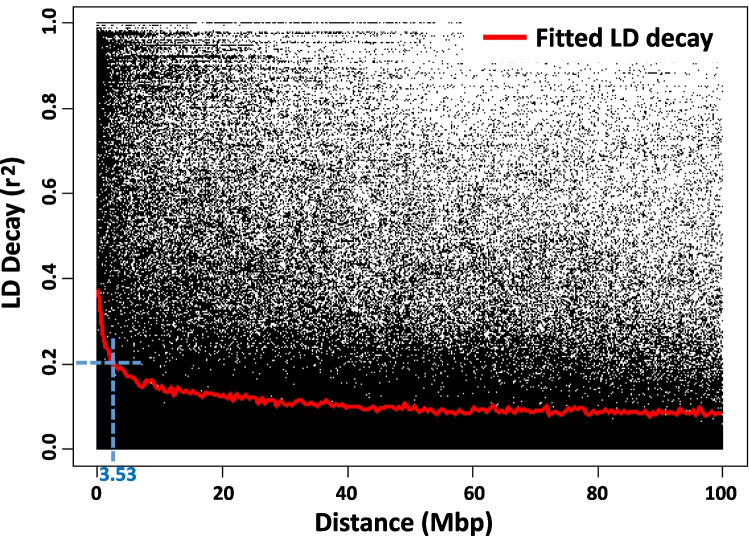
Linkage disequilibrium (LD) decay plots of *r*^2^ over physical distance in megabase pairs (Mbp) on chromosomes. The locally weighted polynomial regression–based (LOESS) fitting curve (red line) illustrates the LD decay. LD decayed to *r*^2^ of 0.2 (blue dotted line) at an approximate distance of 3.53 Mbp

The number of 532 SNP markers complied with defined conditions for credible associations (Table 2, fifth column). Based on the fitted model, LD decayed at 3.53 Mbp for the whole genome at the cut-off *r*^2^ = 0.2. (Fig. 5); therefore, ± 3.53 Mbp was used to establish confidence intervals for trait-associated regions (MTA). In the study, 88 MTA were identified on 13 chromosomes: 1A, 1B, 1D, 2A, 2B, 3A, 3B, 3D, 4A, 4B, 6A, 7A, and 7D. The intervals whose physical positions overlapped were collapsed into 23 QTL (Table 2). It should be noted that in the case of putative detected translocations on chromosomes 1B, 3D, and 4A, the designated intervals were much longer than those defined by the critical *r*^2^ = 0.2 value (Table 2). The detailed information about the markers with the highest significance revealed in each QTL has been provided in Supplementary Table S5. Additionally, the genes located nearest to the most significant markers on reference genome were covered by Supplementary Table S6. Among the most significant markers, there are canonical resistance genes, including those encoding proteins with leucine-rich repeat (LRR) domains. Additionally, many other genes are present in these regions, some of which encode regulatory and signaling components that may contribute to the overall defense response.

**Table 2:** Summary of detected QTL associated with resistance of winter wheat cultivars at seedling stage to *Puccinia triticina* isolates (Pt1–Pt18)

No	QTL	Chromosomal location^1^	Trait^2^	Total number of SNPs detected in the QTL	Marker with highest phenotypic explanation					*Lr* gene postulation
					Name^3^	Position (Mbp)	pFDR†	***R***^2^‡	Resistance allele	
1	*QLr.ihar-1A.1*	1A:57,430,000–61490000	Pt12	1	1202359_10#*	57.963	3.27	0.07	G	
2	*QLr.ihar-1A.2*	1A:117,630,000–124690000	Pt5	1	4990320_6#	121.156	2.44	< 0.01	C	
3	*QLr.ihar-1A.3*	1A:558,400,000–5664600000	Pt4	1	7353014_8#*	562.933	5.44	0.17	T	
			Pt6	1	7353014_8*	562.933	3.65	0.18	T	
4	*QLr.ihar-1B.1*	1B:5,850,000–222820000	Pt10	1	992751_26	39.618	2.59	< 0.01	C	*Lr26*
			Pt11	1	992751_26	39.618	2.1	< 0.01	C	
			Pt12	48	992751_26*	39.618	3.78	< 0.01	C	
			Pt14	21	992751_26	39.618	2.76	0.03	C	
			Pt17	49	1229852_12#*	65.555	5.37	0.17	T	
			Pt18	11	1695427_11	27.228	2.04	0.11	A	
					1695427_29	27.228	2.04	0.11	G	
					3029920_5	39.766	2.04	0.11	C	
					1695183_35	106.857	2.04	0.11	G	
			Pt5	3	4988984_56*	26.159	3.17	0.1	G	
5	*QLr.ihar-1D.1*	1D:1,980,000–9040000	Pt5	1	2265008_47#*	5.512	3.1	0.13	C	
6	*QLr.ihar-1D.2*	1D:75,600,000–82630000	Pt5	1	1205744_31#	79.095	2.44	0.07	G	
7	*QLr.ihar-1D.3*	1D:100,660,000–105150000	Pt14	1	2256740_47	100.662	2.04	0.1	T	
			Pt5	2	2256740_47#	100.662	2.9	0.11	T	
8	*QLr.ihar-2A.1*	2A:715,830,000–723160000	Pt10	1	3029582_37	719.361	2.36	0.12	T	
			Pt13	1	3029582_37	719.361	2	0.03	T	
			Pt18	1	3029582_37	719.361	2.45	0.1	T	
			Pt7	1	3029582_37#	719.361	2.66	0.07	T	
9	*QLr.ihar-2A.2*	2A:516,790,000–523850000	Pt17	1	4990464_34#	520.318	2.17	< 0.01	G	
10	*QLr.ihar-2B.1*	2B:733,580,000–740640000	Pt15	1	11313457_18	737.109	2.65	0.08	C	
			Pt16	1	11313457_18#*	737.109	3.32	0.06	C	
			Pt2	1	11313457_18	737.109	2.38	0.09	C	
11	*QLr.ihar-2B.2*	2B:780,730,000–787790000	Pt3	1	1138981_8*	784.264	3.97	0.27	T	
			Pt4	1	1138981_8#*	784.264	9.67	0.28	T	
			Pt6	1	1138981_8*	784.264	4.66	0.27	T	
12	*QLr.ihar-3A.1*	3A:719,810,000–726870000	Pt1	1	1208992_44	723.339	2.39	0.16	C	
			Pt3	1	1208992_44	723.339	3	0.18	C	
			Pt4	1	1208992_44#*	723.339	9.52	0.36	C	
			Pt6	1	1208992_44*	723.339	3.59	0.2	C	
13	*QLr.ihar-3B.1*	3B:801,700,000–803170000	Pt4	2	19483790_46#*	803.17	7.43	< 0.01	G	
			Pt3	1	19483790_46	803.17	2.53	< 0.01	G	
			Pt6	1	19483790_46*	803.17	3.67	< 0.01	G	
14	*QLr.ihar-3B.2*	3B:819,850,000–826910000	Pt1	1	4412018_37	823.382	2.34	0.19	A	
			Pt3	1	4412018_37	823.382	2.48	0.22	A	
			Pt4	1	4412018_37#*	823.382	4.26	0.21	A	
			Pt6	1	4412018_37	823.382	2.9	0.22	A	
15	*QLr.ihar-3D.1*	3D:596,990,000–613610000	Pt1	33	986749_51*	612.131	3.41	0.22	G	*Lr24*
					986749_53*	612.131	3.41	0.22	T	
			Pt3	34	986749_51*	612.131	4.09	0.25	G	
					986749_53*	612.131	4.09	0.25	T	
			Pt4	33	986749_51#*	612.131	19.62	0.51	G	
					986749_53#*	612.131	19.62	0.51	T	
			Pt6	34	1237949_8*	597.272	4.67	0.23	A	
16	*QLr.ihar-4A.1*	4A:713,610,000–742980000	Pt1	2	1200918_31*	726.102	4.1	0.29	A	*Lr28*
			Pt10	2	1111878_14	726.102	2	0.16	C	
			Pt11	20	2262019_15	729.433	2.12	0.22	A	
			Pt12	23	1111878_14*	726.223	3.34	0.17	C	
			Pt14	14	2264577_44	728.104	2.82	0.14	G	
			Pt15	28	3957935_29*	742.248	4.89	0.23	C	
					5324598_40*	742.248	4.89	0.23	G	
			Pt16	27	3534027_37#*	730.004	8.09	0.26	C	
			Pt17	8	6049552_52*	723.337	3.73	0.24	A	
			Pt18	22	1200918_31*	726.102	5.05	0.14	A	
			Pt2	2	1200918_31*	726.102	7.81	0.34	A	
			Pt5	23	2264577_44*	728.104	3.17	0.27	G	
			Pt8	20	2264577_44*	728.104	6.24	0.43	G	
17	*QLr.ihar-4B.1*	4B:577,550,000–584610000	Pt10	1	986849_35	581.08	2.77	0.11	C	
			Pt13	1	986849_35	581.08	2.77	0.04	C	
			Pt14	1	986849_35	581.08	2.04	0.11	C	
			Pt18	1	986849_35	581.08	2.96	0.11	C	
			Pt7	1	986849_35#*	581.08	4.09	0.08	C	
18	*QLr.ihar-6A.1*	6A:612,840,000–619900000	Pt7	1	3958202_40#	616.369	2.6	0.1	G	
19	*QLr.ihar-7A.1*	7A:630,000–7690000	Pt16	1	1093652_8#	4.155	2.99	0.17	C	
			Pt3	1	1093652_8	4.155	2.59	0.29	C	
20	*QLr.ihar-7A.2*	7A:10,660,000–17720000	Pt15	1	1223836_11#	14.188	2.99	0.25	A	
			Pt16	1	1223836_11	14.188	2.56	0.18	A	
21	*QLr.ihar-7D.1*	7D:170,000–3340000	Pt1	3	11313356_7	0.166	2.58	0.23	C	
			Pt10	1	3954902_8	5.341	2	0.14	G	
			Pt15	6	3954902_8*	5.341	4.89	0.2	G	
			Pt16	7	3954902_8#*	5.341	6.88	0.21	G	
			Pt17	1	3954902_8	5.341	2.09	0.2	G	
			Pt18	2	3954902_8*	5.341	3.39	0.18	G	
			Pt2	4	3954902_8	5.341	4	0.31	G	
			Pt5	1	3954902_8	5.341	2.44	0.22	G	
			Pt8	1	3954902_8	5.341	2.86	0.37	G	
22	*QLr.ihar-7D.2*	7D:15,380,000–22440000	Pt15	1	2248508_28*	18.911	3.13	0.21	T	
			Pt16	1	2248508_28#*	18.911	3.82	0.21	T	
			Pt1	1	2248508_28	18.911	2.13	0.28	T	
			Pt2	1	2248508_28*	18.911	3.12	0.33	T	
23	*QLr.ihar-7D.3*	7D:634,160,000–641220000	Pt17	1	5412064_12#	637.686	2.55	0.13	G	

^1^Chromosome name and interval in base pairs

^2^Resistance to certain *Pt* isolate

^3^The core ID of each DArTseq clone supplemented with the SNP position within the clone sequence

^#^The most significant marker in the QTL interval which was used further for gene postulation in cultivars

^†^False discovery rate

^‡^Explained phenotypic variation of the trait

*Markers with significance at pFDR 0.001

Each QTL contained 1–12 MTAs associated with different traits (reaction to *Pt* isolates Pt1–Pt18). QTL *QLr.ihar-1B.1*, *QLr.ihar-7D.1*, and *QLr.ihar-4A.1* were associated with the largest number of traits, 7, 9, and 12, respectively (Table 2). Most of the QTL were represented by one or two SNP markers. However, *QLr.ihar-7D.1* contained up to seven SNPs significant for Pt16. QTL identified for putative translocations *QLr.ihar-1B.1*, *QLr.ihar-3D.1*, and *QLr.ihar-4A.1* were represented up to several dozen SNPs (Table 2). The same SNP was declared as the most significant for several MTAs detected within the interval, with the exception of *QLr.ihar-1B.1*, *QLr.ihar-3D.1*, *QLr.ihar-4A.1*, and *QLr.ihar-7D.1*.

For 14 QTL, the most significant markers could explain phenotypic variation for reaction to *P. triticina* isolates up to 17%. Larger phenotypic variation in resistance to *P. triticina* isolates in the range 21–28% was explained by *QLr.ihar-2B.2*, *QLr.ihar-3B.2*, *QLr.ihar-4A.1*, *QLr.ihar-7A.2*, *QLr.ihar-7D.1*, and *QLr.ihar-7D.2*. Two QTL, *QLr.ihar-3A.1* and *QLr.ihar-3D.1*, comprised markers that could explain phenotypic variation for reaction to *P. triticina* isolates, 36 and 51%, respectively. In the case of *QLr.ihar-3D.1*, two SNP markers, 986,749–51 and 986,749–53, with the highest determination coefficient were derived from the same DArT clone (Table 2). Figure S1 provides a graphical representation of the raw GWAS results.

### Resistance gene postulation

Based on the defined *Lr* gene postulation criteria (see above), only *QLr.ihar-1B.1*, *QLr.ihar-3D.1*, and *QLr.ihar-4A.1* comprised markers with correct localization for genes represented by Lr-Tc-NILs, i.e., *Lr26*, *Lr24*, and *Lr28*, respectively. The other 20 QTL showed markers for cultivars that did not correspond to any known gene represented by the differential set and were localized on chromosomes 1A (three QTL), 1D (three), 2A (two), 2B (two), 3A, 3B (two), 4B, 6A, 7A (two), and 7D (three) (Table 2). From among 143 cultivars, 51 harbored one to seven resistance loci (including two markers for *Lr24* determined as *QLr.ihar-3D.1*). The cultivar Elixer contained seven resistance loci including *Lr24* and *Lr28*. The resistance genes *Lr24*, *Lr26*, and *Lr28* were detected in 9, 12, and 22 cultivars, respectively (Table S7). The other single resistance loci were detected for up to 14 cultivars (*vide QLr.ihar-7A.2*). Resistant cultivars to all *Pt* isolates used in the study harbored a different set of resistance loci: Lear (*Lr28* only), Capo (*Lr28* and *QLr.ihar-7A.2*), Lithium (*Lr24*, 1 A-3 and *QLr.ihar-2B.2*), Waxy (*Lr24*, *QLr.ihar-1A.3*, and *QLr.ihar-3B.1*), Desamo (*Lr24*, *QLr.ihar-1A.3*, *QLr.ihar-2B.2*, and *QLr.ihar-3B.2*), Xantippe (*Lr24*, *QLr.ihar-1A.3*, *QLr.ihar-2B.2*, and *QLr.ihar-3A.1*), Caro (*Lr24*, *QLr.ihar-1A.3*, *QLr.ihar-2B.2*, *QLr.ihar-3B.1*, and *QLr.ihar-3B.2*), Memory (*QLr.ihar-1A.1*, *QLr.ihar-1A.2*, *QLr.ihar-1 A.3*, *QLr.ihar-2B.2*, and *QLr.ihar-3A.1*), and Tentation (*Lr24*, *QLr.ihar-1A.3*, *QLr.ihar-2B.2*, *QLr.ihar-3A.1*, and *QLr.ihar-3B.2*) (Table S7).

## Discussion

Identification of both known and novel loci involved in resistance processes in currently used cultivars is one of the most effective strategies for managing resistance breeding programs and crop protection. In our study, we used 181 wheat genotypes, including 143 modern cultivars that are highly or moderately resistant to leaf rust. These cultivars were developed by 38 breeding companies from 11 European countries and are intended to represent a substantial part of the Central and West European genetic pool. The GWAS was designed not only to identify leaf rust resistance loci in the studied varieties but also to facilitate the rapid utilization of effective resistance loci in breeding programs. This approach is often challenging when the source materials are landraces, old varieties, or synthetic wheats due to the time-consuming process of eliminating negative traits linked with the desired trait (Bohra et al. 2022). Most GWAS on wheat typically utilize these materials (Kertho et al. 2015; Jighly et al. 2016; Pasam et al. 2017; Sapkota et al. 2019; Kaur et al. 2023).

Among six distinct genetic groups identified in our study, not surprisingly a separate Group I comprised entirely Thatcher near-isogenic lines (NILs) with known *Lr* resistance genes, highlighting a distinct genetic cluster focused on specific genetic material (leaf rust differential set). This is not surprising because all the NILs have all the same Thatcher background and are spring type lines. Interestingly, group IV, which contains only MV Lucilla and line LrB(PI268316), and group V, consisting solely of the *Lr* differential line Gatcher, highlight unique genetic backgrounds. This may indicate specific breeding lines or historical introductions with limited distribution, as noted in studies by Le Couviour et al. (2011), where certain genotypes remained isolated due to unique agronomic traits or specific breeding objectives. However, the majority of cultivars were assigned to three groups (II, III, and VI) revealed by structure analysis, but they were clustered closely together in the PCoA. Furthermore, these cultivars did not cluster by geographical origin or breeding organization, suggesting a widespread exchange of genetic material across Europe (Bentley et al. 2014). This trend suggests that while genetic variability remains, it is increasingly shaped by selective breeding for key agronomic traits rather than traditional geographical differentiation. The clustering of wheat cultivars from breeding companies in genetic analyses is a common phenomenon (Novoselović et al. 2016; El‐Esawi et al., 2018; Kiss et al. 2021). In the present study, linkage disequilibrium (LD) decayed to *r*^2^ = 0.2 at approximately 3.53 Mbp, a value that is broadly comparable to several previous GWAS in wheat. Kaur et al. (2023) observed a genome-wide LD decay of about 1.5 Mbp in a set of 365 hexaploid wheat accessions, whereas Delfan et al. (2023) noted that LD decay in their Iranian bread wheat panel ranged between ~ 2.4 Mbp in the A genome and ~ 5.4 Mbp in the B genome at a similar *r*^2^ cut-off. However, such variation in LD decay across different studies likely reflects underlying differences in germplasm composition, breeding history, marker densities, and population structure of the panels used.

In our study, we identified 88 MTAs that grouped into 23 QTL for seedling resistance to 18 *P. triticina* isolates, which were not evenly distributed across the wheat genome. Chromosomes 1B, 2B, and 7D harbored the highest number of resistance-associated loci. This is consistent with several QTL-rich clusters (QRCs) for leaf rust resistance recently reviewed by Tong et al. (2024), validating our results and highlighting genomic regions consistently associated with resistance across diverse studies. *QLr.ihar-1B.1* (5.85–222.82 Mb) overlaps with multiple QRCs on chromosome 1B, including 1B-I, 1B-II, and 1B-III, encompassing known resistance genes such as *Lr26* and *Lr46*. *QLr.ihar-1D-1* corresponds to QRC 1D-I, which harbors major genes such as *Lr21*, *Lr60*, and *Lr42* and several quantitative resistance loci. *QLr.ihar-2B.1* and *QLr.ihar-2B.2* (733.58–787.79 Mb) align with QRC 2B-XV, while *QLr.ihar-3D.1* (596.99–613.61 Mb) corresponds to QRC 3D-I, albeit with some positional differences. *QLr.ihar-4A.1* (713.61–742.98 Mb) matches QRC 4 A-I, and *QLr.ihar-7D.1* and *QLr.ihar-7D.2* (0.17–22.44 Mb) align with QRC 7D-I, known to harbor *Lr34* (Tong et al. 2024).

Most of the QTL identified in our study do not correspond to any R genes represented in the Tc-NIL-Lr set carrying known *Lr* resistance genes. Based on the correspondence between phenotype/genotype information from Lr-Tc-NILs and the AM panel, as well as the chromosomal locations of known resistance loci recently summarized by Tong et al. (2024), three QTL likely correspond to known *Lr* genes.

*QLr.ihar-1B.1* was collocated with *Lr*26, a resistance gene derived from rye (*Secale cereale*) cv. Petkus and located on the 1BL/1RS translocation in wheat (De Froidmont 1998; Ren et al. 2023). *QLr.ihar-1B.1* spanned a length of approximately 200 Mbp on the chromosome, which is consistent with the putative translocation on 1BS. Based on the identified variants of markers covered by this QTL, the 12 wheat cultivars are believed to possess *Lr*26 (Table S7), confirming its utility in some breeding programs across Europe due to its multiple benefits, particularly in conferring resistance to diseases such as leaf rust, stem rust, and stripe rust (Šrámková et al. 2010; Bartoš et al. 2004; Ambrozková et al. 2002).

The *QLr.ihar-3D.1* physical location covers the *Lr24*/*Sr24* locus, derived from *Agropyron elongatum* (Schachermayr et al. 1995) and known as two distinct translocations, 1BS/3 Ag in cv. Amigo and 3DL/3 Ag in cv. Agent (Uhrin et al. 2008). The length of *QLr.ihar-3D.1* is approximately 44 Mbp. As long as it is localized on 3DL, it is believed to coincide with the 3DL/3 Ag introgression carrying *Lr24*. Virulence to *Lr24* has been reported in European *P. triticina* populations (Mesterházy et al. 2000; Czajowski et al. 2011), reducing its effectiveness. It is less common in Western European countries (Serfling et al. 2011; Fontyn et al. 2022), which is also reflected by the rather low number (nine) of wheat cultivars tested in our study (Table S7).

The location of *QLr.ihar-4A.1* is consistent with the *Lr*28 locus derived from *Aegilops speltoides* (Bipinraj et al. 2011) and spans approximately 29 Mbp. The 22 wheat cultivars analyzed in this study possessed *QLr.ihar-4A.1* (Table S7), confirming rather moderate adoption of the postulated *Lr28* gene among European wheat cultivars (Fontyn et al. 2022).

Beside the postulated *Lr* genes, several detected QTL such as *QLr.ihar-1A.3*, *QLr.ihar-1D.1*, *QLr.ihar-7A.2*, and *QLr.ihar-7D.2* overlap with the location of other leaf rust resistance QTL or *Lr* genes (Tong et al. 2024). However, further fine mapping, haplotype analysis, and allelism tests would be needed to conclusively determine whether these are indeed separate QTL or allelic variants of the same locus.

*QLr.ihar-1A.3* is located in the chromosomal interval on the long arm of 1A, which is also physically occupied by *QLr.ipbb-1A.2* (Genievskaya et al. 2020; Tong et al. 2024). *QLr.ipbb-1A.2* reported by Genievskaya et al. (2020) was effective against leaf rust at both seedling and adult plant stages. This QTL explained 16% of the phenotypic variation, which is remarkably similar to the explanatory power of *QLr.ihar-1A.3*.

The *QLr.ihar-1D.1* region coincides with the location of the major genes *Lr42* and *Lr60* (Hiebert et al. 2008; Lin et al. 2022). The *Lr42* gene, which confers all-stage resistance, was identified from accession TA2450 in a collection of the wild relative of wheat *Aegilops tauschii* Coss. It was transferred into the bread wheat variety ‘Century’ and subsequently released as the germplasm line KS91 WGRC11 in 1991 (Cox et al. 1994). This line has been widely utilized in CIMMYT’s wheat breeding programs (Basnet et al. 2013, 2014). Although the gene’s high utility suggests that it may have been incorporated into European wheat varieties, no specific information confirming this is available. The same holds for the *Lr60*, which was identified from the line V860 of the Watkins collection Dyck (1994) and represents a useful diversification of the genetic resources available in wheat for development of leaf rust resistant cultivars, but there is no information on its incorporation into European wheat breeding programs. Therefore, we can only speculate on the presence of *Lr42* and *Lr60* in nine cultivars of the analyzed wheat panel (Table S7).

*QLr.ihar-1D.1* is located in the chromosomal interval where other QTL were identified, i.e., *QLr.spa-1D* (Bokore et al. 2020), *QLr.tam-1D* (Basnet et al. 2014), *QLrs.B22-1D*, and *QLr.B22-1D* (Naz et al. 2008). The *QLr.ihar-1D.1* QTL identified in the present study explained 13% of the phenotypic variation for seedling resistance to *P. triticina*. This is relatively low compared to *QLr.spa-1D* identified by Bokore et al. (2020), which explained up to 35% of the phenotypic variation for adult plant leaf rust resistance across environments. Similarly, *QLr.tam-1D* reported by Basnet et al. (2014) accounted for 24–35% of the variation in leaf rust severity. The *QLr.B22-1D* QTL characterized by Naz et al. (2008) had an even larger effect, explaining 35–49% of the phenotypic variation for seedling resistance. However, the *QLrs.B22-1D* QTL for seedling resistance in the same study had a more modest effect of 15% explained variation, which is closer to our findings for *QLr.ihar-1D.1*.

*QLr.ihar-7A.2* is located in the interval on the short arm of chromosome 7A, which is also the physical location of *QLr.mna-7AL* (Tsilo et al. 2014; Tong et al. 2024). However, *QLr.mna-7AL* was associated with adult plant resistance to leaf rust across multiple environments and explained up to 8.1% of the phenotypic variation for leaf rust severity (Tsilo et al. 2014). This is much lower than the 25% explained by *QLr.ihar-7A.2*.

*QLr.ihar-7D.2* was mapped to the short arm of chromosome 7D, spanning the physical interval 15.38–22.44 Mbp. It was associated with resistance to four *P. triticina* isolates at the seedling stage. The most significant marker explained up to 33% of the phenotypic variation. Similarly, *QLr.jki-7D.1* was also located on chromosome 7DS, with the peak marker positioned at 16.1 Mbp (Rollar et al. 2021; Tong et al. 2024). This QTL was detected for adult plant resistance in field trials and explained 28% of the phenotypic variance for leaf rust severity. *QLr.ihar-7D.2* was identified for seedling resistance, while *QLr.jki-7D.1* conferred adult plant resistance, and no information is available on its seedling resistance.

Among the detected QTL, 14 cover the markers with the significance at pFDR with a cut-off of 0.001: *QLr.ihar1A.1*, *QLr.ihar1A.3*, *QLr.ihar-1B.1*, *QLr.ihar-1D.1*, *QLr.ihar-2B.1*, *QLr.ihar-2B.2*, *QLr.ihar-3A.1*, *QLr.ihar-3B.1*, *QLr.ihar-3B.2*, *QLr.ihar-3D.1*, *QLr.ihar-4A.1*, *QLr.ihar-4B.1*, *QLr.ihar-7D.1*, and *QLr.ihar-7D.2*. Nevertheless, those QTL do not always correspond to those explaining the highest phenotypic variance. This discrepancy arises from several factors. Small-effect loci may be obscured by background noise, leading to associations that lack significant biological relevance (Walkowiak et al. 2020). Additionally, low allele frequencies and weak linkage disequilibrium can hinder the practical impact of high-significance loci. Moreover, hidden genetic variation and epistatic interactions contribute to this disconnect, underscoring the complexities of the genetic landscape (Ling et al. 2018). While significant *q*-values indicate potential associations, they do not guarantee meaningful phenotypic explanations.

The 16 of detected QTL potentially represent novel leaf rust resistance loci, as the physical intervals in which they occur do not overlap with the locations of previously described quantitative or qualitative resistance loci (Tong et al. 2024). These include *QLr.ihar-1A.1*, *QLr.ihar-1A.2*, *QLr.ihar-1D.2*, *QLr.ihar-1D.3*, *QLr.ihar-2A.1*, *QLr.ihar-2A.2*, *QLr.ihar-2B.1*, *QLr.ihar-2B.2*, *QLr.ihar-3A.1*, *QLr.ihar-3B.1*, *QLr.ihar-3B.2*, *QLr.ihar-4B.1*, *QLr.ihar-6A.1*, *QLr.ihar-7A.1*, *QLr.ihar-7D.1*, and *QLr.ihar-7D.3*. Of these, *QLr.ihar-2B.2*, *QLr.ihar-3A.1*, *QLr.ihar-3B.2*, *QLr.ihar-7A.1*, *QLr.ihar-7D.1*, and *QLr.ihar-7D.2* explain a significant proportion of the phenotypic variance (over 20%) and may be of breeding interest after validation across different environmental conditions and confirmation of their relevance in adult plant resistance.

This validation is crucial, as seedling resistance identified under controlled conditions is not always expressed as adult plant resistance in the field (Ellis et al. 2014). Furthermore, the effectiveness of QTL can be influenced by genetic background and environmental factors (Singh et al. 2016).

The varying levels of virulence among isolates suggest the presence of diverse resistance genes in the wheat panel studied (Table S7). In the study, nine cultivars resistant to all Pt isolates (Capone, Caroll, Desamo, Lear, Lithium, Memory, Tentation, Waxy, and Xantippe) do not share a common resistance pattern locus. This may suggest the presence of resistance in these cultivars is conditioned by diverse and undetected resistance loci. Furthermore, 113 cultivars were resistant to at least one isolate, but the 23 detected QTLs were present in only 51 genotypes. This indicates that for 62 cultivars, resistance to *P. triticina* is conditioned by entirely different resistance loci not detected in our study.

Several factors may explain the limited QTL detection in this GWAS of leaf rust–resistant wheat. The highly repetitive and polyploid nature of the wheat genome exacerbates reference genome biases. In our study, GWAS relies on a single reference genome, which may fail to represent the structural variants diversity such as insertions, deletions, and copy number variations. Walkowiak et al. (2020) highlighted how the reliance on limited genomic references can lead to gaps in understanding genetic variation across diverse wheat lines. Moreover, regions absent from the reference genome remain unaccounted for, which can lead to underestimated associations for certain QTLs. The development of wheat pangenomes offers a more comprehensive representation of genetic diversity, significantly aiding in capturing structural variants and improving the accuracy of QTL detection. This concept aligns with Ling et al. (2018), who underline the importance of genomic diversity in enhancing GWAS methodologies. Low allele frequency of some resistance loci can limit detection due to reduced statistical power (Kaur et al. 2023). Small-effect loci are easily masked by background noise (Pasam et al. 2017), and epistatic interactions, crucial for resistance, are often missed by standard GWAS analyses (Alseekh et al. 2021). Weak linkage disequilibrium (LD) between markers and resistance loci, especially for small-effect loci, further hinders detection (Gangurde et al. 2022). Population structure can confound results due to allele frequency differences between subgroups (Pritchard et al. 2000). Phenotyping errors can also impact GWAS outcomes by misclassifying resistant/susceptible lines (Sing et al. 2022). Finally, the complex, polygenic nature of leaf rust resistance, influenced by environmental factors, challenges the sensitivity of GWAS methods (Iqbal et al. 2022).

Identifying both known and novel loci for leaf rust resistance in wheat is crucial for advancing breeding programs. In our study, using GWAS, a number of MTAs were identified, some linked to known resistance genes and others potentially new. This research offers insights into the genetic diversity of resistance in European wheat, supporting more targeted breeding strategies.

## Supplementary Information

Below is the link to the electronic supplementary material.

TABLE S1 (DOCX 32.1 KB)

TABLE S2 (DOCX 22.1 KB)

TABLE S3 (XLSX 25.5 KB)

TABLE S4 (DOCX 32.2 KB)

TABLE S5 (XLSX 12.4 KB)

TABLE S6 (XLSX 23.4 KB)

TABLE S7 (XLSX 29.0 KB)

FIG S1 (PPTX 19.6 MB)

## Data Availability

Data generated or analysed during this study are included in the main text article and its supplementary files. Data not included are available from the correspondent author on reasonable request.
