# Supplementary material for: Genome-wide association study of seedling leaf rust resistance in European winter wheat cultivars
Source: J Appl Genet. 2025 Jun 9;66(4):853–69. doi: 10.1007/s13353-025-00976-2 (PMC12616754; doi:10.1007/s13353-025-00976-2)
Supplement: Supplementary file 1 — TABLE S1 (DOCX 32.1 KB) [file 13353_2025_976_MOESM1_ESM.docx]

Genome-wide association study of seedling leaf rust resistance in European winter wheat cultivars

Paweł Cz. Czembor, Urszula Piechota, Jie Song, Dariusz Mańkowski, Magdalena Radecka-Janusik, Dominika Piaskowska, Piotr Słowacki, Andrzej Kilian

Supplementary table S1. Wheat cultivars used in association mapping for leaf rust resistance genes.

| **No** | **Object number** | **Cultivar** | **Breeder/Maintener** | **Country** |
| --- | --- | --- | --- | --- |
| 1 | 101 | Addict | Lemaire Deffontaines | France |
| 2 | 103 | Agape | SIS - Società Italiana Sementi | Italy |
| 3 | 1 | Akteur | Deutsche Saatveredelung AG | Germany |
| 4 | 2 | Alcazar | DANKO Hodowla Roślin sp. z o. o. | Poland |
| 5 | 3 | Arkadia | DANKO Hodowla Roślin sp. z o. o. | Poland |
| 6 | 4 | Arktis | Deutsche Saatveredelung AG | Germany |
| 7 | 113 | Armada | Nickerson International Research SNC | France |
| 8 | 115 | Artagnan | Nickerson International Research SNC | France |
| 9 | 5 | Artist | Deutsche Saatveredelung AG | Germany |
| 10 | 6 | Askalon | Nordsaat Saatzucht GmbH | Germany |
| 11 | 116 | Astardo | Saatzucht Donau Ges.m.b.H. & CoKG | Austria |
| 12 | 7 | Astoria | Poznańska Hodowla Roślin sp. z o.o. | Poland |
| 13 | 8 | Bagou | Saaten Union Recherche | France |
| 14 | 9 | Baletka | RAGT 2n | France |
| 15 | 10 | Bamberka | Hodowla Roślin Strzelce sp. z o. o. Grupa IHAR | Poland |
| 16 | 11 | Banderola | DANKO Hodowla Roślin sp. z o. o. | Poland |
| 17 | 12 | Batuta | DANKO Hodowla Roślin sp. z o. o. | Poland |
| 18 | 13 | Belenus | RAGT 2n | France |
| 19 | 123 | Belepi | Blackman Agriculture | Great Britain |
| 20 | 14 | Bockris | Strube Research GmbH & Co. KG | Germany |
| 21 | 15 | Bogatka | DANKO Hodowla Roślin sp. z o. o. | Poland |
| 22 | 16 | Boomer | Dieckmann GmbH & Co KG | Germany |
| 23 | 17 | Bystra | RAGT 2n | France |
| 24 | 131 | Calumet | Florimond Desprez Veuve et Fils | France |
| 25 | 133 | Capone | Limagrain GmbH | Germany |
| 26 | 135 | Caroll | B.V. Landbouwbureau Wiersum | The Netherlands |
| 27 | 136 | Celebration | Deutsche Saatveredelung AG | Germany |
| 28 | 140 | Cocoon | Secobra Recherches | France |
| 29 | 146 | Desamo | Syngenta Seeds GmbH | Germany |
| 30 | 150 | Diderot | Secobra Recherches | France |
| 31 | 18 | Dorota | RAGT 2n | France |
| 32 | 152 | Edelrun | Saatzucht LFS | Austria |
| 33 | 19 | Elipsa | Limagrain Belgium N.V. | Belgium |
| 34 | 157 | Elixer | W. von Borries-Eckendorf GmbH & Co. Kommanditgesellschaft | Germany |
| 35 | 161 | Ennsio | Saatzucht LFS | Austria |
| 36 | 162 | Eperon | Saaten Union Recherche | France |
| 37 | 163 | Eriwan | Saatzucht LFS | Austria |
| 38 | 164 | Eron* | Saatzucht LFS | Austria |
| 39 | 166 | Estevan | Saatzucht LFS | Austria |
| 40 | 20 | Estivus | Strube Research GmbH & Co. KG | Germany |
| 41 | 169 | Event | Saatzucht Josef Breun GmbH & Co. KG | Germany |
| 42 | 21 | Fakir | Syngenta Seeds GmbH | Germany |
| 43 | 174 | Fermi | Florimond Desprez Veuve et Fils | France |
| 44 | 22 | Fidelius | Saatzucht Donau Ges.m.b.H. & CoKG | Austria |
| 45 | 23 | Figura | DANKO Hodowla Roślin sp. z o. o. | Poland |
| 46 | 24 | Forkida | DANKO Hodowla Roślin sp. z o. o. | Poland |
| 47 | 25 | Forum | Nordsaat Saatzucht GmbH | Germany |
| 48 | 26 | Fregata | Hodowla Roślin Strzelce sp. z o. o. Grupa IHAR | Poland |
| 49 | 27 | Garantus | RAGT 2n | France |
| 50 | 183 | Genius | Nordsaat Saatzucht GmbH | Germany |
| 51 | 186 | Gordian | Syngenta Seeds GmbH | Germany |
| 52 | 187 | Granamax | Agri Obtentions SA | France |
| 53 | 188 | Grapeli | Agri Obtentions SA | France |
| 54 | 28 | Henrik | Limagrain GmbH | Germany |
| 55 | 189 | Hermann | Limagrain GmbH | Germany |
| 56 | 190 | Heros | Sejet Planteforaedling | Denmark |
| 57 | 193 | Ionesco | Secobra Recherches | France |
| 58 | 29 | Jantarka | DANKO Hodowla Roślin sp. z o. o. | Poland |
| 59 | 30 | Jenga | Ackermann Saatzucht GmbH & Co. KG | Germany |
| 60 | 196 | Joker | Deutsche Saatveredelung AG | Germany |
| 61 | 197 | Julius | KWS Lochow GmbH | Germany |
| 62 | 31 | Kampana | DANKO Hodowla Roślin sp. z o. o. | Poland |
| 63 | 200 | Kantao | Serasem | France |
| 64 | 32 | Kepler | Limagrain GmbH | Germany |
| 65 | 33 | Kobiera | Małopolska Hodowla Roślin - HBP sp. z o. o. | Poland |
| 66 | 34 | Kohelia | Małopolska Hodowla Roślin - HBP sp. z o. o. | Poland |
| 67 | 35 | Kranich | Lantmännen SW Seed GmbH | Germany |
| 68 | 36 | Kredo | Nordsaat Saatzucht GmbH | Germany |
| 69 | 37 | Kris | RAGT 2n | France |
| 70 | 41 | KWS Ozon | KWS Lochow GmbH | Germany |
| 71 | 203 | KWS Cashel | KWS Lochow GmbH | Germany |
| 72 | 38 | KWS Dacanto | KWS Lochow GmbH | Germany |
| 73 | 204 | KWS Erasmus | KWS Lochow GmbH | Germany |
| 74 | 205 | KWS Kielder | KWS Lochow GmbH | Germany |
| 75 | 39 | KWS Livius | KWS Lochow GmbH | Germany |
| 76 | 40 | KWS Magic | KWS Lochow GmbH | Germany |
| 77 | 206 | KWS Pius | KWS Lochow GmbH | Germany |
| 78 | 208 | Lahertis | Strube Research GmbH & Co. KG | Germany |
| 79 | 42 | Lavantus | Strube Research GmbH & Co. KG | Germany |
| 80 | 211 | Lear | Limagrain GmbH | Germany |
| 81 | 43 | Legenda | Poznańska Hodowla Roślin sp. z o.o. | Poland |
| 82 | 44 | Linus | RAGT 2n | France |
| 83 | 212 | Lithium | Adrien Momont et Fils | France |
| 84 | 45 | Look | Dieckmann GmbH & Co KG | Germany |
| 85 | 46 | Ludwig | DANKO Hodowla Roślin sp. z o. o. | Poland |
| 86 | 47 | Markiza | Hodowla Roślin Strzelce sp. z o. o. Grupa IHAR | Poland |
| 87 | 221 | Matheo | Deutsche Saatveredelung AG | Germany |
| 88 | 48 | Meister | RAGT 2n | France |
| 89 | 224 | Memory | Secobra Recherches | France |
| 90 | 49 | Meteor | Syngenta Seeds GmbH | Germany |
| 91 | 50 | Mewa | DANKO Hodowla Roślin sp. z o. o. | Poland |
| 92 | 51 | Mikula | Małopolska Hodowla Roślin - HBP sp. z o. o. | Poland |
| 93 | 227 | Modern | Adrien Momont et Fils | France |
| 94 | 52 | Mulan | Nordsaat Saatzucht GmbH | Germany |
| 95 | 53 | Muszelka | DANKO Hodowla Roślin sp. z o. o. | Poland |
| 96 | 54 | Muza | Małopolska Hodowla Roślin - HBP sp. z o. o. | Poland |
| 97 | 213 | MV Lucilla | Prebázis Kft | Hungary |
| 98 | 55 | Naridana | Poznańska Hodowla Roślin sp. z o.o. | Poland |
| 99 | 56 | Natula | Poznańska Hodowla Roślin sp. z o.o. | Poland |
| 100 | 233 | Nocibe | Syngenta Seeds | France |
| 101 | 57 | Nutka | Hodowla Roślin Strzelce sp. z o. o. Grupa IHAR | Poland |
| 102 | 58 | Olivin | RAGT 2n | France |
| 103 | 59 | Operetka | Nickerson International Research SNC | France |
| 104 | 60 | OstkaStrzelecka | Hodowla Roślin Strzelce sp. z o. o. Grupa IHAR | Poland |
| 105 | 61 | Ostroga | DANKO Hodowla Roślin sp. z o. o. | Poland |
| 106 | 62 | Oxal | RAGT 2n | France |
| 107 | 238 | Pamier | Lantmännen SW Seed GmbH | Germany |
| 108 | 63 | Patras | Secobra Recherches | France |
| 109 | 64 | Pengar | W. von Borries-Eckendorf GmbH & Co. Kommanditgesellschaft | Germany |
| 110 | 242 | Pionier | Secobra Recherches | France |
| 111 | 65 | Platin | Strube Research GmbH & Co. KG | Germany |
| 112 | 66 | Praktik | RAGT 2n | France |
| 113 | 67 | Rapsodia | RAGT 2n | France |
| 114 | 248 | Rebell | RAGT 2n | France |
| 115 | 252 | RGT Djoko | RAGT 2n | France |
| 116 | 68 | Rywalka | Hodowla Roślin Strzelce sp. z o. o. Grupa IHAR | Poland |
| 117 | 69 | Sailor | Secobra Recherches | France |
| 118 | 70 | Satyna | Małopolska Hodowla Roślin - HBP sp. z o. o. | Poland |
| 119 | 264 | Scout | Senova Ltd | Great Britain |
| 120 | 71 | Skagen | W. von Borries-Eckendorf GmbH & Co. Kommanditgesellschaft | Germany |
| 121 | 72 | Smaragd | SW Seed GmbH | Germany |
| 122 | 73 | Smuga | DANKO Hodowla Roślin sp. z o. o. | Poland |
| 123 | 267 | Solky | Caussade Semences | France |
| 124 | 270 | Sorokk | Caussade Semences | France |
| 125 | 74 | Speedway | Nordsaat Saatzucht GmbH | Germany |
| 126 | 271 | Stadium | Adrien Momont et Fils | France |
| 127 | 75 | Sukces | Hodowla Roślin Strzelce sp. z o. o. Grupa IHAR | Poland |
| 128 | 274 | Tabasco | W. von Borries-Eckendorf GmbH & Co. Kommanditgesellschaft | Germany |
| 129 | 276 | Tentation | Lemaire Deffontaines | France |
| 130 | 277 | Terroir | Florimond Desprez Veuve et Fils | France |
| 131 | 278 | Thalys | Syngenta Seeds | France |
| 132 | 279 | Tobak | W. von Borries-Eckendorf GmbH & Co. Kommanditgesellschaft | Germany |
| 133 | 76 | Tonacja | Hodowla Roślin Strzelce sp. z o. o. Grupa IHAR | Poland |
| 134 | 77 | Torrild | W. von Borries-Eckendorf GmbH & Co. Kommanditgesellschaft | Germany |
| 135 | 78 | Tulecka | Poznańska Hodowla Roślin sp. z o.o. | Poland |
| 136 | 79 | Türkis | Lantmännen SW Seed GmbH | Germany |
| 137 | 80 | Turnia | Małopolska Hodowla Roślin - HBP sp. z o. o. | Poland |
| 138 | 285 | Waxy | Dieckmann GmbH & Co KG | Geramny |
| 139 | 81 | Wydma | Hodowla Roślin Smolice sp. z o. o. Grupa IHAR | Poland |
| 140 | 289 | Xantippe | Sejet Planteforaedling | Denmark |
| 141 | 291 | Zappa | Ackermann Saatzucht GmbH & Co. KG | Germany |
| 142 | 82 | Zawisza | Hodowla Roślin Smolice sp. z o. o. Grupa IHAR | Poland |
| 143 | 83 | Zyta | Hodowla Roślin Strzelce sp. z o. o. Grupa IHAR | Poland |

* - not registered line SE 302/10
