# Supplementary material for: Genome-wide association study of seedling leaf rust resistance in European winter wheat cultivars
Source: J Appl Genet. 2025 Jun 9;66(4):853–69. doi: 10.1007/s13353-025-00976-2 (PMC12616754; doi:10.1007/s13353-025-00976-2)
Supplement: Supplementary file 2 — TABLE S2 (DOCX 22.1 KB) [file 13353_2025_976_MOESM2_ESM.docx]

Genome-wide association study of seedling leaf rust resistance in European winter wheat cultivars

Paweł Cz. Czembor, Urszula Piechota, Jie Song, Dariusz Mańkowski, Magdalena Radecka-Janusik, Dominika Piaskowska, Piotr Słowacki, Andrzej Kilian

Supplementary table S2. Lines with known resistance genes used in association mapping for leaf rust resistance genes.

| **No** | **Object number** | **Lr gene** | **Pedigree** | **Identity number** |
| --- | --- | --- | --- | --- |
| 1 | 501 | *LrTc* | Thatcher | RL 6101 |
| 2 | 748 | *Lr1* | Tc*6/Centenario | RL 6003 |
| 3 | 749 | *Lr2a* | Tc*6/Webster | RL 6016 |
| 4 | 750 | *Lr2b* | Tc*6/Carina | RL 6019 |
| 5 | 751 | *Lr2c* | Tc*6/Loros | RL 6047 |
| 6 | 752 | *Lr3a* | Tc*6/Democrat | RL 6002 |
| 7 | 753 | *Lr3bg* | Tc* 6/Bage | RL 6042 |
| 8 | 754 | *Lr3ka* | Tc*6/Klein Aniversario | RL 6007 |
| 9 | 755 | *Lr9* | Transfer/Tc*6 *Aegilops.umbellulata* | RL 6010 |
| 10 | 756 | *Lr10* | Tc*6/Exchange | RL 6004 |
| 11 | 757* | *Lr11* | Tc*2/Hussar | RL 6053 |
| 12 | 760 | *Lr14a* | Selkirk/Tc*6 (*T. turgidum*) | RL 6013 |
| 13 | 761 | *Lr14b* | Tc*6/Mario Escobar | RL 6006 |
| 14 | 762 | *Lr15* | Tc*6/W1483 | RL 6052 |
| 15 | 763 | *Lr16* | Tc*6/Exchange | RL 6005 |
| 16 | 764 | *Lr17a* | Klein Lucero/Tc*6 | RL 6008 |
| 17 | 765 | *Lr18* | Tc*7/Afrika 43 (*T. timopheevii*) | RL 6009 |
| 18 | 766 | *Lr19* | Tc*7 Transloc.4-*Agropyron elongatum* | RL 6040 |
| 19 | 767 | *Lr20* | Tc*6/Jimmer | RL 6092 |
| 20 | 768 | *Lr21* | Tc*6RL5406 *Ae. Squarrosa v.mayeri* | RL 6043 |
| 21 | 770 | *Lr23* | Lee 310/Tc*6 | RL 6012 |
| 22 | 771 | *Lr24* | Tc*6/Agent (*Agropyron elongatum*) | RL 6064 |
| 23 | 772 | *Lr25* | Tc*6/Transec (*Secale cereale*) | RL 6084 |
| 24 | 773 | *Lr26* | Tc*6/St-1-25 (*Secale cereale*) | RL 6078 |
| 25 | 528 | *Lr27+31* | Gatcher | Gatcher |
| 26 | 774 | *Lr28* | Tc*6/C-77-1 (*Aegilops speltoides*) | RL 6079 |
| 27 | 775 | *Lr29* | Tc*6/CS7D-Ag + 11 *(Agropyron elongatum*) | RL 6080 |
| 28 | 776 | *Lr30* | Tc*6/Terenzio | RL 6049 |
| 29 | 777 | *Lr32* | Tc*6/3/ *Aegilops squarrosa* | RL 6086 |
| 30 | 778 | *Lr33* | Tc*6/PI 58548-1 | RL 6057 |
| 31 | 536 | *Lr36* | ER 84018 *(Aegilops speltoides)* | ER 84018 |
| 32 | 782 | *Lr38* | Tc*6/T7 Kohn *(Thinopyrum intermedium)* | RL 6097 |
| 33 | 783* | *Lr44* | Tc*6*/T.spelta* | RL 6147 |
| 34 | 784 | *Lr52* | Tc*6/V336 | RL 6107 |
| 35 | 538 | *Lr63* | Tc*6/TMR5-J14-12-24 (*T. monococcum*) | RL 6137 |
| 36 | 540 | *Lr64* | Tc*6/8404 (*T. turgidum ssp. dicoccoides*) | RL 6149 |
| 37 | 785 | *LrB(Carina)* | Tc*6/Carina | RL 6051 |
| 38 | 546 | *LrB(PI268316)* | Tc*6/PI268316 | RL 6061 |

* – no DArTseq data available
