# Supplementary material for: Genome-wide association study of seedling leaf rust resistance in European winter wheat cultivars
Source: J Appl Genet. 2025 Jun 9;66(4):853–69. doi: 10.1007/s13353-025-00976-2 (PMC12616754; doi:10.1007/s13353-025-00976-2)
Supplement: Supplementary file 4 — TABLE S4 (DOCX 32.2 KB) [file 13353_2025_976_MOESM4_ESM.docx]

Genome-wide association study of seedling leaf rust resistance in European winter wheat cultivars

Paweł Cz. Czembor, Urszula Piechota, Jie Song, Dariusz Mańkowski, Magdalena Radecka-Janusik, Dominika Piaskowska, Piotr Słowacki, Andrzej Kilian

Supplementary table S4. The groups (subpopulations) of cultivars/lines determined in population structure analysis.

| **Group** | **Cultivar/line** | **Breeder/pedigree** | **Country/ID** |
| --- | --- | --- | --- |
| 1 | Lr2b | Tc*6/Carina | RL 6019 |
| 1 | Lr38 | Tc*6/T7 Kohn (Thinopyrum intermedium) | RL 6097 |
| 1 | Lr2c | Tc*6/Loros | RL 6047 |
| 1 | Lr16 | Tc*6/Exchange | RL 6005 |
| 1 | LrTc | Thatcher | RL 6101 |
| 1 | Lr14a | Selkirk/Tc*6 (T. turgidum) | RL 6013 |
| 1 | Lr9 | Transfer/Tc*6 Aegilops.umbellulata | RL 6010 |
| 1 | Lr63 | Tc*6/TMR5-J14-12-24 (T. monococcum) | RL 6137 |
| 1 | Lr3bg | Tc* 6/Bage | RL 6094 |
| 1 | Lr52 | Tc*6/V336 | RL 6107 |
| 1 | Lr10 | Tc*6/Exchange | RL 6004 |
| 1 | Lr14b | Tc*6/Mario Escobar | RL 6006 |
| 1 | Lr28 | Tc*6/C-77-1 (Aegilops speltoides) | RL 6079 |
| 1 | Lr30 | Tc*6/Terenzio | RL 6049 |
| 1 | LrB(Carina) | Tc*6/Carina | RL 6051 |
| 1 | Lr33 | Tc*6/PI 58548-1 | RL 6057 |
| 1 | Lr2a | Tc*6/Webster | RL 6016 |
| 1 | Lr32 | Tc*6/3/ Aegilops squarrosa | RL 6086 |
| 1 | Lr3 | Tc*6/Democrat | RL 6002 |
| 1 | Lr15 | Tc*6/W1483 | RL 6052 |
| 1 | Lr25 | Tc*6/Transec (Secale cereale) | RL 6084 |
| 1 | Lr64 | Tc*6/8404 (T. turgidum ssp. dicoccoides) | RL 6149 |
| 1 | Lr18 | Tc*7/Afrika 43 (T. timopheevii) | RL 6009 |
| 1 | Lr1 | Tc*6/Centenario | RL 6003 |
| 1 | Lr21 | Tc*6RL5406 Ae. Squarrosa v.mayeri | RL 6043 |
| 1 | Lr3ka | Tc*6/Klein Aniversario | RL 6042 |
| 1 | Lr23 | Lee 310/Tc*6 | RL 6012 |
| 1 | Lr36 | ER 84018 (Aegilops speltoides) | ER 84018 |
| 1 | Lr26 | Tc*6/St-1-25 (Secale cereale) | RL 6078 |
| 1 | Lr17 | Klein Lucero/Tc*6 | RL 6008 |
| 1 | Lr20 | Tc*6/Jimmer | RL 6092 |
| 1 | Lr19 | Tc*7 Transloc.4-Agropyron elongatum | RL 6040 |
| 1 | Lr24 | Tc*6/Agent (Agropyron elongatum) | RL 6064 |
| 1 | Lr29 | Tc*6/CS7D-Ag + 11 (Agropyron elongatum) | RL 6080 |
| 2 | Meteor | Syngenta Seeds GmbH | Germany |
| 2 | Rywalka | Hodowla Roślin Strzelce sp. z o. o. Grupa IHAR | Poland |
| 2 | Türkis | Lantmännen SW Seed GmbH | Germany |
| 2 | Legenda | Poznańska Hodowla Roślin sp. z o.o. | Poland |
| 2 | Pamier | Lantmännen SW Seed GmbH | Germany |
| 2 | Kranich | Lantmännen SW Seed GmbH | Germany |
| 2 | Arktis | Deutsche Saatveredelung AG | Germany |
| 2 | Naridana | Poznańska Hodowla Roślin sp. z o.o. | Poland |
| 2 | Fregata | Hodowla Roślin Strzelce sp. z o. o. Grupa IHAR | Poland |
| 2 | Muza | Małopolska Hodowla Roślin - HBP sp. z o. o. | Poland |
| 2 | Mikula | Małopolska Hodowla Roślin - HBP sp. z o. o. | Poland |
| 2 | Kohelia | Małopolska Hodowla Roślin - HBP sp. z o. o. | Poland |
| 2 | OstkaStrzelecka | Hodowla Roślin Strzelce sp. z o. o. Grupa IHAR | Poland |
| 2 | Skagen | W. von Borries-Eckendorf GmbH & Co. Kommanditgesellschaft | Germany |
| 2 | Julius | KWS Lochow GmbH | Germany |
| 2 | Kobiera | Małopolska Hodowla Roślin - HBP sp. z o. o. | Poland |
| 2 | Smaragd | SW Seed GmbH | Germany |
| 2 | Praktik | RAGT 2n | France |
| 2 | Eriwan | Saatzucht LFS | Austria |
| 2 | Estevan | Saatzucht LFS | Austria |
| 2 | Zawisza | Hodowla Roślin Smolice sp. z o. o. Grupa IHAR | Poland |
| 2 | Event | Saatzucht Josef Breun GmbH & Co. KG | Germany |
| 2 | Garantus | RAGT 2n | France |
| 2 | Wydma | Hodowla Roślin Smolice sp. z o. o. Grupa IHAR | Poland |
| 2 | Natula | Małopolska Hodowla Roślin - HBP sp. z o. o. | Poland |
| 2 | Fakir | Syngenta Seeds GmbH | Germany |
| 2 | Akteur | Deutsche Saatveredelung AG | Germany |
| 2 | Ludwig | DANKO Hodowla Roślin sp. z o. o. | Poland |
| 2 | Mewa | DANKO Hodowla Roślin sp. z o. o. | Poland |
| 2 | Agape | SIS - Società Italiana Sementi | Italy |
| 2 | Memory | Secobra Recherches | France |
| 2 | Eron* | Saatzucht LFS | Austria |
| 2 | Astardo | Saatzucht Donau Ges.m.b.H. & CoKG | Austria |
| 2 | Smuga | DANKO Hodowla Roślin sp. z o. o. | Poland |
| 2 | Edelrun | Saatzucht LFS | Austria |
| 2 | Baletka | RAGT 2n | France |
| 3 | Alcazar | DANKO Hodowla Roślin sp. z o. o. | Poland |
| 3 | Kampana | DANKO Hodowla Roślin sp. z o. o. | Poland |
| 3 | Muszelka | DANKO Hodowla Roślin sp. z o. o. | Poland |
| 3 | Kris | RAGT 2n | France |
| 3 | Jantarka | DANKO Hodowla Roślin sp. z o. o. | Poland |
| 3 | Henrik | Limagrain GmbH | Germany |
| 3 | Banderola | DANKO Hodowla Roślin sp. z o. o. | Poland |
| 3 | Mulan | Nordsaat Saatzucht GmbH | Germany |
| 3 | Waxy | Dieckmann GmbH & Co KG | Geramny |
| 3 | Rapsodia | RAGT 2n | France |
| 3 | Meister | RAGT 2n | France |
| 3 | Boomer | Dieckmann GmbH & Co KG | Germany |
| 3 | Linus | RAGT 2n | France |
| 3 | Bystra | RAGT 2n | France |
| 3 | Kepler | Limagrain GmbH | Germany |
| 3 | Bockris | Strube Research GmbH & Co. KG | Germany |
| 3 | Jenga | Ackermann Saatzucht GmbH & Co. KG | Germany |
| 3 | KWS Pius | KWS Lochow GmbH | Germany |
| 3 | Olivin | RAGT 2n | France |
| 3 | Nocibe | Syngenta Seeds | France |
| 3 | Operetka | Nickerson International Research SNC | France |
| 3 | Ionesco | Secobra Recherches | France |
| 3 | KWS Erasmus | KWS Lochow GmbH | Germany |
| 3 | Belenus | RAGT 2n | France |
| 3 | Cocoon | Secobra Recherches | France |
| 3 | Artagnan | Nickerson International Research SNC | France |
| 3 | Eperon | Saaten Union Recherche | France |
| 3 | Lithium | Adrien Momont et Fils | France |
| 3 | Tulecka | Poznańska Hodowla Roślin sp. z o.o. | Poland |
| 3 | Astoria | Poznańska Hodowla Roślin sp. z o.o. | Poland |
| 3 | Pionier | Secobra Recherches | France |
| 3 | Arkadia | DANKO Hodowla Roślin sp. z o. o. | Poland |
| 3 | Figura | DANKO Hodowla Roślin sp. z o. o. | Poland |
| 3 | Lahertis | Strube Research GmbH & Co. KG | Germany |
| 3 | Caroll | B.V. Landbouwbureau Wiersum | The Netherlands |
| 3 | Tabasco | W. von Borries-Eckendorf GmbH & Co. Kommanditgesellschaft | Germany |
| 3 | Zappa | Ackermann Saatzucht GmbH & Co. KG | Germany |
| 3 | KWS Livius | KWS Lochow GmbH | Germany |
| 3 | Bagou | Saaten Union Recherche | France |
| 3 | Askalon | Nordsaat Saatzucht GmbH | Germany |
| 3 | Platin | Strube Research GmbH & Co. KG | Germany |
| 3 | Celebration | Deutsche Saatveredelung AG | Germany |
| 3 | KWS Dacanto | KWS Lochow GmbH | Germany |
| 3 | Xantippe | Sejet Planteforaedling | Denmark |
| 3 | Sukces | Hodowla Roślin Strzelce sp. z o. o. Grupa IHAR | Poland |
| 3 | KWS Ozon | KWS Lochow GmbH | Germany |
| 3 | Tonacja | Hodowla Roślin Strzelce sp. z o. o. Grupa IHAR | Poland |
| 3 | Genius | Nordsaat Saatzucht GmbH | Germany |
| 3 | Zyta | Hodowla Roślin Strzelce sp. z o. o. Grupa IHAR | Poland |
| 3 | Look | Dieckmann GmbH & Co KG | Germany |
| 3 | Hermann | Limagrain GmbH | Germany |
| 3 | Granamax | Agri Obtentions SA | France |
| 3 | Tentation | Lemaire Deffontaines | France |
| 3 | Ostroga | DANKO Hodowla Roślin sp. z o. o. | Poland |
| 3 | Elipsa | Limagrain Belgium N.V. | Belgium |
| 3 | Bamberka | Hodowla Roślin Strzelce sp. z o. o. Grupa IHAR | Poland |
| 3 | Artist | Deutsche Saatveredelung AG | Germany |
| 3 | Joker | Deutsche Saatveredelung AG | Germany |
| 3 | Sorokk | Caussade Semences | France |
| 3 | Lavantus | Strube Research GmbH & Co. KG | Germany |
| 3 | Oxal | RAGT 2n | France |
| 3 | Estivus | Strube Research GmbH & Co. KG | Germany |
| 3 | Patras | Secobra Recherches | France |
| 3 | Rebell | RAGT 2n | France |
| 3 | Batuta | DANKO Hodowla Roślin sp. z o. o. | Poland |
| 3 | Diderot | Secobra Recherches | France |
| 3 | Solky | Caussade Semences | France |
| 3 | Satyna | Małopolska Hodowla Roślin - HBP sp. z o. o. | Poland |
| 3 | Torrild | W. von Borries-Eckendorf GmbH & Co. Kommanditgesellschaft | Germany |
| 3 | Ennsio | Saatzucht LFS | Austria |
| 3 | Armada | Nickerson International Research SNC | France |
| 3 | Kantao | Serasem | France |
| 3 | Calumet | Florimond Desprez Veuve et Fils | France |
| 3 | Grapeli | Agri Obtentions SA | France |
| 3 | Nutka | Hodowla Roślin Strzelce sp. z o. o. Grupa IHAR | Poland |
| 3 | Modern | Adrien Momont et Fils | France |
| 3 | KWS Kielder | KWS Lochow GmbH | Germany |
| 3 | Sailor | Secobra Recherches | France |
| 3 | Bogatka | DANKO Hodowla Roślin sp. z o. o. | Poland |
| 3 | Forum | Nordsaat Saatzucht GmbH | Germany |
| 3 | Dorota | RAGT 2n | France |
| 3 | KWS Magic | KWS Lochow GmbH | Germany |
| 3 | Turnia | Małopolska Hodowla Roślin - HBP sp. z o. o. | Poland |
| 3 | Markiza | Hodowla Roślin Strzelce sp. z o. o. Grupa IHAR | Poland |
| 3 | Matheo | Deutsche Saatveredelung AG | Germany |
| 3 | Thalys | Syngenta Seeds | France |
| 3 | Forkida | DANKO Hodowla Roślin sp. z o. o. | Poland |
| 3 | Speedway | Nordsaat Saatzucht GmbH | Germany |
| 3 | Fidelius | Saatzucht Donau Ges.m.b.H. & CoKG | Austria |
| 3 | KWS Cashel | KWS Lochow GmbH | Germany |
| 4 | MV Lucilla | Prebázis Kft | Hungary |
| 4 | LrB(PI268316) | Tc*6/PI268316 | RL 6061 |
| 5 | Lr27+31 | Gatcher | Gatcher |
| 6 | Stadium | Adrien Momont et Fils | France |
| 6 | Terroir | Florimond Desprez Veuve et Fils | France |
| 6 | Tobak | W. von Borries-Eckendorf GmbH & Co. Kommanditgesellschaft | Germany |
| 6 | Addict | Lemaire Deffontaines | France |
| 6 | Belepi | Blackman Agriculture | Great Britain |
| 6 | Pengar | W. von Borries-Eckendorf GmbH & Co. Kommanditgesellschaft | Germany |
| 6 | Scout | Senova Ltd | Great Britain |
| 6 | Heros | Sejet Planteforaedling | Denmark |
| 6 | Lear | Limagrain GmbH | Germany |
| 6 | Kredo | Nordsaat Saatzucht GmbH | Germany |
| 6 | Fermi | Florimond Desprez Veuve et Fils | France |
| 6 | Capone | Limagrain GmbH | Germany |
| 6 | Gordian | Syngenta Seeds GmbH | Germany |
| 6 | Desamo | Syngenta Seeds GmbH | Germany |
| 6 | Elixer | W. von Borries-Eckendorf GmbH & Co. Kommanditgesellschaft | Germany |
| 6 | RGT Djoko | RAGT 2n | France |

* - not registered line SE 302/10
