# Supplementary material for: Genome-wide association study of seedling leaf rust resistance in European winter wheat cultivars
Source: J Appl Genet. 2025 Jun 9;66(4):853–69. doi: 10.1007/s13353-025-00976-2 (PMC12616754; doi:10.1007/s13353-025-00976-2)
Supplement: Supplementary file 8 — FIG S1 (PPTX 19.6 MB) [file 13353_2025_976_MOESM8_ESM.pptx]

## Slide 1
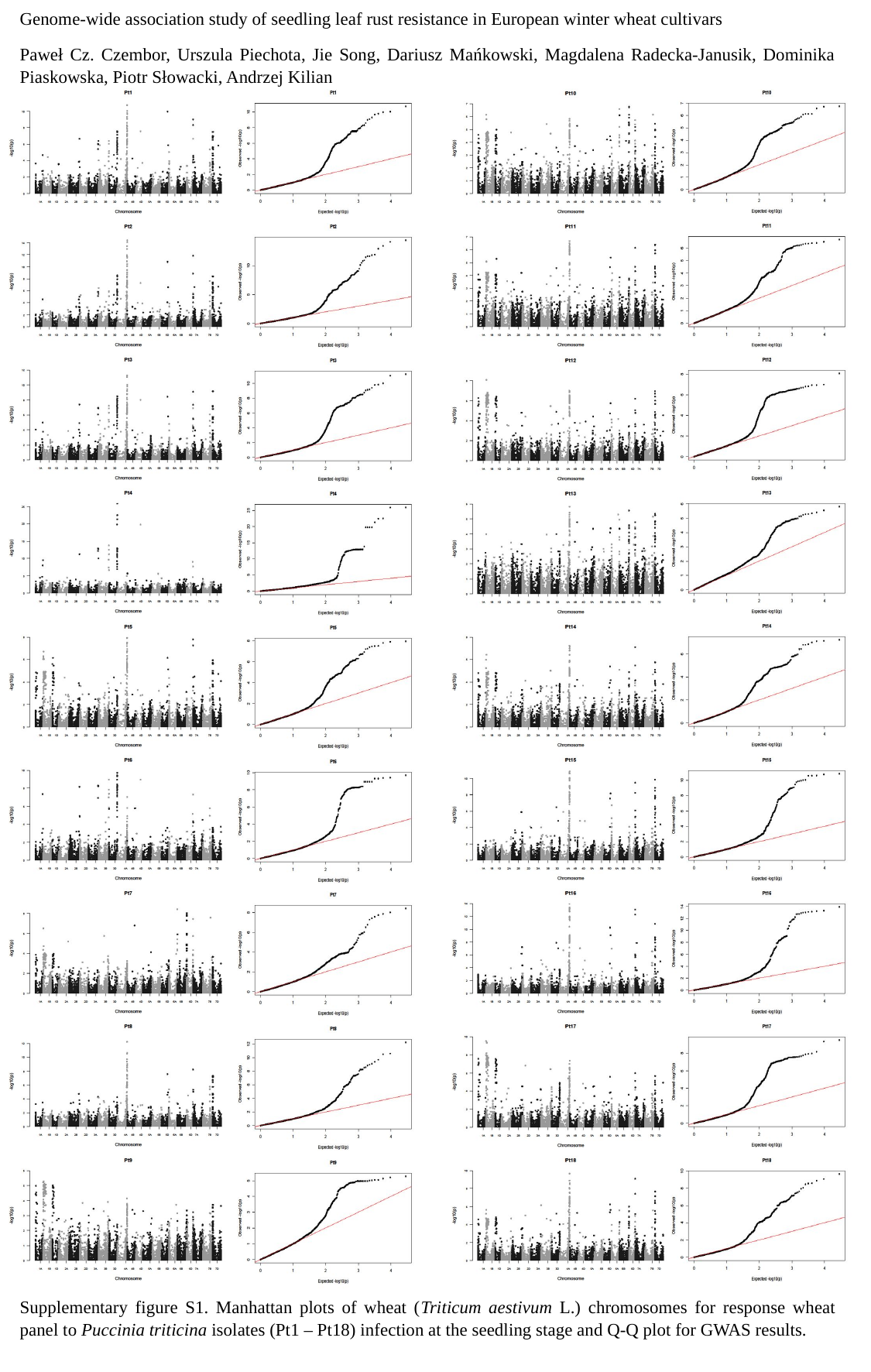

Genome-wide association study of seedling leaf rust resistance in European winter wheat cultivars
Paweł Cz. Czembor, Urszula Piechota, Jie Song, Dariusz Mańkowski, Magdalena Radecka-Janusik, Dominika Piaskowska, Piotr Słowacki, Andrzej Kilian
Supplementary figure S1. Manhattan plots of wheat (Triticum aestivum L.) chromosomes for response wheat panel to Puccinia triticina isolates (Pt1 – Pt18) infection at the seedling stage and Q-Q plot for GWAS results.
